# Altered Interhemispheric Functional Connectivity Associated With Early Verbal Fluency Decline After Deep Brain Stimulation in Parkinson’s Disease

**DOI:** 10.3389/fnagi.2022.799545

**Published:** 2022-04-01

**Authors:** Bei Luo, Wenwen Dong, Lei Chang, Chang Qiu, Yue Lu, Dongming Liu, Chen Xue, Li Zhang, Weiguo Liu, Wenbin Zhang, Jun Yan

**Affiliations:** ^1^Department of Functional Neurosurgery, The Affiliated Brain Hospital of Nanjing Medical University, Nanjing, China; ^2^Department of Neurosurgery, The Affiliated Brain Hospital of Nanjing Medical University, Nanjing, China; ^3^Department of Radiology, The Affiliated Brain Hospital of Nanjing Medical University, Nanjing, China; ^4^Department of Geriatric Neurology, The Affiliated Brain Hospital of Nanjing Medical University, Nanjing, China; ^5^Department of Neurology, The Affiliated Brain Hospital of Nanjing Medical University, Nanjing, China

**Keywords:** Parkinson’s disease, deep brain stimulation, verbal fluency, voxel-mirrored homotopic connectivity, resting state functional magnetic resonance

## Abstract

**Background:**

Patients with Parkinson’s disease (PD) experience a decline in verbal fluency (VF) immediately after undergoing deep brain stimulation (DBS) of the subthalamic nucleus (STN). This phenomenon is thought to be related to surgical microlesions.

**Purpose:**

We investigated the alterations in interhemispheric functional connectivity after STN-DBS in PD patients. We also evaluated the correlation between these changes and decreased VF scores.

**Method:**

Overall, 30 patients with PD were enrolled in the study. Resting-state functional magnetic resonance imaging scans were performed twice, once before and once after DBS, in PD patients. Voxel-mirrored homotopic connectivity (VMHC) was applied in order to evaluate the synchronicity of functional connectivity between the hemispheres.

**Result:**

After undergoing STN-DBS, PD patients demonstrated reduced VMHC value in the posterior cerebellum lobe, angular gyrus, precuneus/posterior cingulate gyrus (PCC), supramarginal gyrus, superior frontal gyrus (SFG) (medial and dorsolateral) and middle frontal gyrus (MFG). In addition, we observed a significant positive correlation between the altered VMHC value in the SFG and MFG and the change of phonemic VF scores.

**Conclusion:**

PD patients demonstrated an interhemispheric coordination disorder in the prefrontal cortex, cerebellum, supramarginal gyrus and DMN after undergoing STN-DBS. The positive correlation between reduced VMHC value in the SFG and MFG and the changes of VF scores provides a novel understanding with regard to the decline of VF after DBS.

## Introduction

Deep brain stimulation (DBS), a widely accepted and effective treatment for mid-to-late-stage Parkinson’s disease (PD), improves the motor symptoms and the quality of life of patients, and reduces complications that are caused by anti-Parkinsonian drugs (Deuschl et al., 2006; Benabid et al., 2009). However, after the deep brain electrodes are implanted into the subthalamic nucleus (STN), PD patients often experience adverse neuropsychological reactions. Although the overall cognitive level of patients after chronic STN-DBS is relatively safe (Witt et al., 2008), a decline in verbal fluency (VF) decline is a common postoperative cognitive side effect (Mikos et al., 2011; Lefaucheur et al., 2012; Borden et al., 2014; Le Goff et al., 2015; Costentin et al., 2019). The specific mechanism behind this side effect remains unclear. Multiple studies have discovered that PD patients demonstrate an immediate decline in VF performance after undergoing DBS surgery, while PD patients who suffered from STN-DBS did not demonstrate any significant change in VF scores under “switched-on” and “switched-off” stimulus conditions (Morrison et al., 2004; Witt et al., 2004). To date, evidence suggests that a decline in language fluency after DBS is more likely related to surgical microlesions than to stimulating-induced reactions.

Advanced cognitive processes require participation of both hemispheres (Sauerwein and Lassonde, 1994). The corpus callosum is thought to contain major interhemispheric pathways (Sauerwein and Lassonde, 1994). Previous studies have demonstrated that people with disconnection or atrophy of the corpus callosum tend to have sensory, motor, and cognitive processing deficits (Dimond, 1979; Sauerwein and Lassonde, 1994; Yaldizli et al., 2014), which illustrates the importance of coordinating between the hemispheres for implementation of high-level complex tasks. One study discovered that atrophy of the corpus callosum affects the performance of VF tasks, highlighting the importance the integrity of the corpus callosum for cognitive information processing related to VF (Pozzilli et al., 1991). Given the importance of inter-hemispheric coordination for VF tasks, we hypothesized that decreased VF in PD patients after DBS may be associated with a deficiency in inter-hemispheric interaction.

Functional magnetic resonance (fMRI), particularly functional connectivity, is an important tool to study the basis of neurological and psychiatric disorders. The resting-state fMRI is able to capture the pattern of fluctuation of blood oxygen levels in resting-state, which has better operability and repeatability, compared to task-state fMRI (Smitha et al., 2019). Functional homotopy is an essential feature of the inner functional structure of the brain, and refers to a high degree of synchronization of spontaneous activity in the corresponding positions of the hemisphere (Zuo et al., 2010; Luo et al., 2015). The homotopic resting-state function connectivity (RSFC) is a good indicator of interhemispheric coordination, and reflects the degree of integration of brain functions, which may be detected through the voxel-mirrored homotopic connectivity (VMHC) method (Hu et al., 2015; Luo et al., 2015). Alterations of homotopic RSFC were discovered in normal aging, as well as neurological and mental illness (Zuo et al., 2010; Guo et al., 2014; Cao et al., 2020; Gan et al., 2020). The VMHC approach has been widely utilized to study neural mechanisms of PD, and has become an important tool to identify changes in inter-hemispheric functional communication (Hu et al., 2015; Li et al., 2018; Gan et al., 2020; Jin et al., 2021).

Herein, we hypothesized that a decline in VF performance after DBS in PD patients may be associated with dysfunction of functional coordination between the hemispheres. Therefore, we analyzed resting-state fMRI data prior to and after STN-DBS surgery among PD patients using the VMHC method to determine changes in homotopic RSFC. Furthermore, we evaluated the correlation between VMHC values of significantly different brain regions before and after DBS and VF scores.

## Materials and Methods

### Participants

The data used in this study is from PD patients who were treated with functional neurosurgery at the Brain Hospital affiliated with Nanjing Medical University. Overall, 37 patients with PD were recruited, all of whom met United Kingdom Parkinson’s Disease Society Brain Bank clinical diagnostic criteria. DBS surgery was carried out on all recruited PD patients after evaluating indications for DBS surgery. The exclusion criteria were as follows: (1) previous neurological disorders and psychiatric history, (2) a history of suffering from non-PD diseases affecting the nervous system (i.e., brain trauma), (3) having taken drugs that affect brain function for six months (i.e., antipsychotics), and (4) contraindications to magnetic resonance examination. All participants were right-handed. This study was granted approval by the Ethics Committee of Brain Hospital affiliated with Nanjing Medical University. All subjects signed written informed consent prior to the start of the experiment.

### Clinical Assessments

The VF test mainly assess spontaneous verbal motor ability, which can be divided into semantic fluency and phonemic fluency. The semantic fluency test asks participants to name as many animals as they can think of in 1 min. Due to the different educational backgrounds of the participants, we chose a Chinese version of the test in order to evaluate phonemic fluency of all subjects. For detailed description, please refer to previous literature (Quan et al., 2015), which has been used in the phonemic fluency test of Chinese people (Liao et al., 2019; Yang et al., 2020). The testing process consists of three phases, including a 30-s baseline, a 60-s task, and a 30-s break after the task. During the task, three Chinese characters (白, 天, and 大, representing white, day and big, respectively) were shown to the testers and each character lasted for 20 s. Next, participants were asked to verbally generate as many phrases or four-character idioms as possible, starting with each given character. The total number of correct animals or words that each participant could say was scored. Patients with PD were evaluated four times using the VF test, including three days before DBS, one day after DBS, one month after DBS, and six months after DBS. At the same time, we also assessed the overall cognitive level of all participants using Montreal Cognitive Assessment (MoCA). In addition, MRI data for PD patients was collected three days before DBS and one day after DBS, which were included in the PD-Pre-DBS and PD-Post-DBS group, respectively. All scales and MRI data were collected after the patient had stopped taking anti-Parkinsonian drugs for more than 12 h, and lacked electrical stimulation. The details of the VF and MoCA assessment were provided in Supplementary Table.

### Surgery

Deep brain stimulation (DBS) surgery was carried out by a single neurosurgeon via a unified surgical procedure in this study. Bilateral STN was chosen as an implant target in all patients. Prior to implanting the stimulation electrode, the STN nucleus single-cell discharge was monitored through the use of OMEGA electrophysiological instrument and the recording electrode. Furthermore, the DBS electrodes (model L301, PINS, Pins Medical Co, China) were implanted after the location was determined. The electrode implantation was completely in accordance with the preoperative target plan, and the electrode position was not adjusted during or after surgery. We did not observe any significant surgical complications on postoperative cranial imaging. The specific position of electrode implantation was shown in the Supplementary Material.

### Image Acquisition

The MRI data were acquired with 1.5 Tesla GE Medical Systems scanner (produced by GE Medical System, Milwaukee, WI) equipped with an eight-channel head coil. Structural images were acquired through the use of 3D magnetization-prepared rapid gradient-echo sequence (MPRAGE) with the following parameters: repetition time (TR) of 11.864 ms, echo time (TE) of 4.932 ms, flip angle (FA) of 20°, number of slices = 112, matrix size = 256 × 256, field of view (FOV) = 152 × 152mm^2^, thickness of 1.4mm, and voxel size of 0.59 × 0.59 × 1.4 mm^3^. Functional images were acquired through the use of a gradient-recalled echo-planar imaging sequence (GRE-EPI) with the following parameters: TR of 2000ms, TE of 40 ms, FA of 90°, FOV = 240 × 240 mm^2^, matrix size = 64 × 64, number of slices = 28, thickness of 3.0mm with no gap, spatial resolution = 3.75 × 3.75 × 3mm^3^, and number of total volumes = 128. During the MRI scans, all participants were instructed to close their eyes, stay relaxed and awake, and not think about anything in particular.

### Data Preprocessing

Resting-state fMRI data preprocessing was carried out by the Data Processing Assistant for resting-State fMRI (DPABI_V4.3^1^) on the MATLAB 2013b platform.^2^ The steps of data preprocessing are briefly described as follows. The first five points were discarded and the remaining 123 images underwent slice-time and motion corrections. Seven PD patients were excluded for exhibiting head movements greater than 3mm or 3 degrees. The individual T1 structure image was co-registered with an average EPI image and segmented into either gray matter or white matter using a new segment and DARTEL segmentation algorithm. Next, the structural images were spatially normalized to the Montreal Neurological Institute (MNI) standard template space, and the transformation information obtained were applied to EPI images. The generated image was then resampled to 3 × 3 × 3mm^3^ and spatially smoothed with a 6 mm full width half maximum Gaussian kernel. The resulting EPI data were linearly trend removed and temporally filtered (0.01–0.10Hz). Next, the nuisance signals were regressed out, including 24 motion parameters, global signals, white matter signals, and cerebrospinal fluid signals using a general linear mode.

### Voxel-Mirrored Homotopic Connectivity Analysis

The VMHC value represents the Pearson correlation coefficient between each voxel’s residual time series, as well as corresponding residual time series in another hemisphere, as described in previous studies (Zuo et al., 2010). First, the normalized T1 images of all participants were averaged in order to generate mean normalized T1 image. The left and right mirror versions of this image were averaged to the group-specific symmetrical T1 template. Then, the normalized T1 image was registered into a specific symmetric template. The transformation information was applied to normalized functional image. The VMHC computation was performed using the DPABI_V4.3 software. Then, Fisher Z transform was performed on correlation values in order to improve normality. The resulting value represents the VMHC value.

### Statistical Analysis

Statistical analysis of demographic and clinical characteristics for PD patients was carried out using SPSS Statistics 22.0 (IBM, Armonk, NY, United States) using repeated measures analysis of variance and following *post hoc t*-test, as appropriate. The paired *t*-test was utilized to identify VMHC differences between the PD-Pre-DBS and the PD-Post-DBS groups with mean framewise displacement (FD) as covariates. All of the above results were corrected by multiple comparisons of the family wise error rate with a voxel *p* < 0.001 and cluster *p* < 0.05 using SPM12 (London, United Kingdom^3^).

### Correlation Analysis

The brain regions with statistically significant differences between PD patients before and after surgery were defined as regions of interest (ROIs). For each PD patient before DBS surgery, the REST software.^4^ was utilized to calculate mean VMHC values for each ROI. The Pearson correlation analysis with SPSS 22.0 software helped calculate the correlation between VMHC values of each ROI, as well as preoperative VF score. In addition, we investigated whether alterations in inter-hemispheric functional connections induced by surgical microlesions correlated with decreased VF scores.

## Results

### Demographic and Clinical Characteristics

Overall, 30 PD patients were included in this study. The demographics of all participants are presented in Table 1. We discovered that MoCA and VF scores of PD patients immediately decreased after surgery, and MoCA scores returned to preoperative levels one month after surgery. However, VF scores were found to still be significantly lower than before.

**TABLE 1:** Demographic and clinical data of all subjects.

	PD (*n* = 30) Mean ± SD	*P*-value
Age (years)	62.27 ± 8.73	-
Sex (male/female)	15/15	-
Education (year)	8.1 ± 3.38	-
Disease duration (year)	8.37 ± 2.86	-
LEDD (mg/d)	787.2 ± 181.89	-
**MoCA score**		
Before DBS	24.70 ± 2.60	0.003^a^*
The first day after DBS	22.37 ± 4.18	
One month after DBS	24.17 ± 2.93	-
Six months after DBS	24.13 ± 2.81	-
**Semantic VF**		
Before DBS	20.20 ± 4.34	< 0.001^a^*
The first day after DBS	14.07 ± 3.95	-
One month after DBS	16.30 ± 3.87	-
Six months after DBS	16.23 ± 4.06	-
**Phonemic VF**		
Before DBS	10.10 ± 2.06	< 0.001^a^*
The first day after DBS	7.37 ± 1.73	
One month after DBS	8.73 ± 2.08	-
Six months after DBS	8.87 ± 1.68	-

*PD, Parkinson’s disease; LEDD, levodopa equivalent daily dose; MoCA, Montreal Cognitive Assessment; VF, verbal fluency; DBS, deep brain stimulation; Mean ± SD, mean ± standard deviation.*

*^a^Repeated measures variance analysis.*

*P < 0.05.

### Voxel-Mirrored Homotopic Connectivity Findings

**FIGURE 1:**
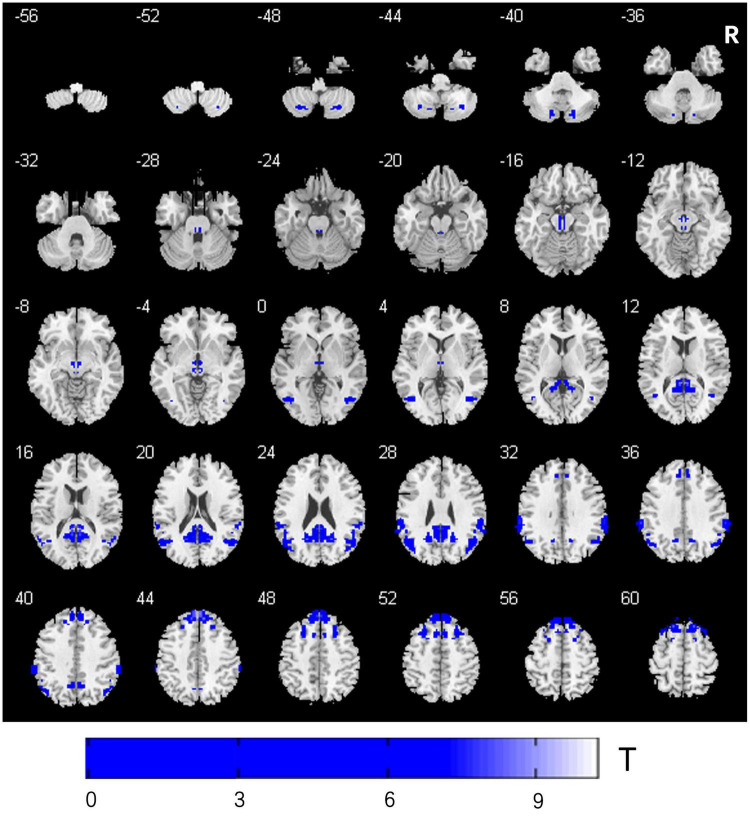
Comparison of VMHC between the PD-Pre-DBS group and PD-Post-DBS group. PD, Parkinson’s disease; DBS, deep brain stimulation; PD-Pre-DBS, three days before DBS, PD-Post-DBS, one day after DBS; VMHC, voxel-mirrored homotopic connectivity; L, left; R, right; Regions showing decreased VMHC in blue; Family wise error correction (voxel *p* < 0.001, cluster *p* < 0.05).

Compared to the PD-Pre-DBS group, the PD-Post-DBS group demonstrated decreased VMHC values in the posterior cerebellum lobe, midbrain, angular gyrus, precuneus/posterior cingulate gyrus (PCC), supramarginal gyrus, superior frontal gyrus (SFG) (medial and dorsolateral) and middle frontal gyrus (MFG). We did not find any increased VMHC value in the PD-Post-DBS group, compared to the PD-Pre-DBS group (see Figure 1 and Table 2).

**TABLE 2:** VMHC differences between the PD-Pre-DBS and PD-Post-DBS group.

	Brain region (AAL)	Cluster size	Peak MNI coordinate			Peak intensity
**PD-Pre-DBS > PD-Post-DBS**						
Cluster 1	Cerebelum_Crus2_R	46	± 18	−75	−39	5.1415
Cluster 2	Midbrain	45	± 3	−15	−9	6.3615
Cluster 3	Angular_R	205	± 45	−66	0	5.3717
Cluster 4	Precuneus_R Cingulum_Post_R	270	± 3	−42	15	9.0389
Cluster 5	SupraMarginal_R	127	± 63	−33	36	6.5664
Cluster 6	Superior Frontal Gyrus (Medial + dorsolateral)	332	± 3	48	42	7.6007
	Frontal_Mid_R					

*PD, Parkinson’s disease; DBS, deep brain stimulation; PD-Pre-DBS, three days before DBS, PD-Post-DBS, one day after DBS; VMHC, voxel-mirrored homotopic connectivity; AAL, anatomical automatic labeling; MNI, Montreal Neurological Institute; Family wise error correction (voxel p < 0.001, cluster p < 0.05).*

### Correlation Analysis

**FIGURE 2:**
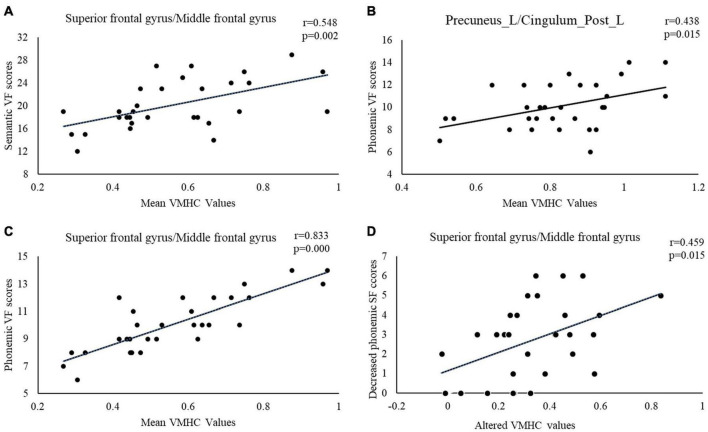
The correlations between VF scores and mean VMHC values in brain regions with statistically significant differences between PD patients before and after surgery. Relationships between: **(A)** Semantic VF and mean VMHC value of superior frontal gyrus and middle frontal gyrus, **(B)** Phonemic VF and mean VMHC value of precuneus/posterior cingulate gyrus, **(C)** Phonemic VF and mean VMHC value of superior frontal gyrus and middle frontal gyrus, and **(D)** Decreased phonemic VF and altered VMHC value in the superior frontal gyrus and middle frontal gyrus. VMHC, voxel-mirrored homotopic connectivity; VF, verbal fluency.

A correlation analysis demonstrated that significant positive correlations were discovered between phonemic VF scores and the VMHC value of the precuneus/PCC (see Figure 2B), SFG and MFG before DBS (see Figure 2C). A significant positive correlation was also seen between semantic VF scores and VMHC value of SFG and MFG (see Figure 2A). Additionally, altered VMHC value in SFG and MFG demonstrated a significant positive correlation with a change in phonemic VF scores (see Figure 2D).

## Discussion

Herein, VMHC was utilized, for the first time and to the best of our knowledge, to study homotopic RSFC changes among PD patients immediately after DBS surgery. Strong and weak homotopic RSFC were interpreted as a tendency to coordinate processing or independent processing in allelic brain regions, respectively (Baldo et al., 2001). In the past, many functional imaging studies have discovered impaired interhemispheric coordination among PD patients (Hu et al., 2015; Zhu et al., 2016; Li et al., 2018; Gan et al., 2021). The focus of this study was to explore changes in functional coordination of homotopic brain regions post-DBS. Our main findings included that PD patients had decreased interhemispheric RSFC in the prefrontal cortex, cerebellum, supramarginal gyrus and default mode network (DMN)-related brain regions. Furthermore, we observed significant positive correlations between the VMHC values of SFG and MFG before DBS and phonemic VF scores in PD patients. The VMHC changes of SFG and MFC induced by DBS surgery were found to be positively correlated with decreased phonemic VF. DBS is able to cause a temporary decline in overall cognitive function among PD patients after surgery. However, in the long run, there was no significant influence on the overall cognitive function. The VF performance of PD patients decreased significantly immediately after DBS, and improved one month later. In the long term, VF performance declined compared to before the surgery, which is consistent with results in previous literature (Lefaucheur et al., 2012; Borden et al., 2014; Le Goff et al., 2015; Costentin et al., 2019).

Our results demonstrated that altered VMHC values were discovered in the prefrontal cortex, including in the SFG and MFG, and in the PD-Pre-DBS group compared to the PD-Post-DBS group. The integrity of executive function or higher cognitive tasks depends on integrity of the structure and function of the frontal lobe (Yuan and Raz, 2014). In addition, increased frontal lobe activity was consistently observed in the resting state fMRI studies of normal adults performing tasks (Yuan and Raz, 2014). The prefrontal lobe is a key area for word comprehension and production (Costafreda et al., 2006). The semantic and phonemic fluency was found to be impaired to varying degrees after partial frontal lobe damage (Baldo and Shimamura, 1998; Thompson-Schill et al., 1998). In addition, impaired VF is considered to be a marker of frontal lobe dysfunction (Baldo and Shimamura, 1998; Baldo et al., 2001). The left side of the brain is the dominant hemisphere and, therefore, the VF is more sensitive to damage to the left prefrontal lobe (Janowsky et al., 1989). However, one study found that VF performance was significantly decreased, regardless of left or right frontal lobe damage (Baldo and Shimamura, 1998). FMRI studies have generally found that the VF task is associated with activation of the frontal and parietal lobes (Vitali et al., 2005; Birn et al., 2010). The decline in VF among patients with PD occurs at initial stages of this illness and is one of the common cognitive changes among PD patients (Henry and Crawford, 2004; Dubois et al., 2007). Pereira et al. (2009) discovered that gray matter density in the frontal lobe, temporal lobe and cerebellum were significantly correlated with semantic fluency scores. The VF performance in PD patients decreased immediately after deep brain electrode implantation. We hypothesized that decreased postoperative VF performance is likely related to decreased homotopic RSFC between the bilateral frontal lobes. Our study discovered that altered VMHC value of SFG and MFG was correlated with changes in phonemic VF scores, which further confirms our speculation.

We also discovered that PD patients had decreased VMHC values in the posterior lobe of the cerebellum after DBS. It is commonly believed that the cerebellum is associated with coordinating voluntary movement, regulating muscle tension and body balance. Research has shown that the cerebellum plays an increasingly important role in the processing of higher cognitive functions, including language, emotion and memory (Hubrich-Ungureanu et al., 2002; De Smet et al., 2013; Starowicz-Filip et al., 2017). A large number of fMRI studies have demonstrated that the right cerebellum was significantly activated during semantic and sentence processing and VF tasks (Hubrich-Ungureanu et al., 2002; Starowicz-Filip et al., 2017; Geva et al., 2021). Hubrich-Ungureanu et al. (2002) applied fMRI in order to check the activation of brain activity in left-handed and right-handed normal volunteers, while carrying out silent VF tasks. When the right-hand volunteers performed a language task, the left fronto-parietal cortex and right cerebellar hemisphere were found to be visibly activated, while the left-hand volunteers were discovered to be visibly activated in the right fronto-parieto-temporal cortex and left cerebellar hemisphere. The volunteers performed VF tasks in a silent state in order to ensure that the brain areas that were activated were due to speech production rather than vocal action. Alexander et al. (2012) discovered that patients with localized lesions of the right cerebellum had lower VF manifestations compared to those with localized lesions of the left cerebellum. These studies have proven the importance of the cerebellum in VF task execution. Therefore, we suggest that interhemispheric coordination disorders of the bilateral cerebellum may be involved in decreased performance of speech fluency, immediately after surgery.

Another important finding in our study was the decrease of VMHC value in the angular gyrus and precuneus/PCC. These brain regions were key brain areas of the DMN, which was associated with cognitive dysfunction among many diseases, including PD and Alzheimer’s disease (AD) (Ding et al., 2014; Liao et al., 2018; Wolters et al., 2019). Liao et al. (2018) demonstrated that the inter-hemispheric RSFC of DMN among AD patients was significantly reduced and that the VMHC peak value of the precuneus was significantly positively correlated with the MoCA score. Therefore, we speculated that the overall cognitive decline of postoperative patients may be related to dysfunction of interhemispheric functional coordination in the DMN brain regions. Additionally, we also observed a reduced interhemispheric synchrony between supramarginal gyrus. The gray matter density of the bilateral supramarginal gyrus is known to be positively correlated with vocabulary knowledge among monolinguals (Lee et al., 2007; Grogan et al., 2012). Bilinguals, relative to monolinguals, have a higher gray matter density in the supramarginal gyrus, and the gray matter density was shown to be positively correlated with vocabulary knowledge (Lee et al., 2007; Grogan et al., 2012). Behaviorally, both vocabulary and VF tasks involve verbal output, and the vocabulary in the sample are significantly correlated with both semantic fluency and phonological production (Lee et al., 2007). It can be concluded from the above studies that VF performance may be related to the density of gray matter in the supramarginal gyrus. Therefore, we hypothesized that the postoperatively homotopic coordination disorder in the supramarginal gyrus observed may be related to the impairment of postoperative speech fluency.

There were several limitations to this study. First, VMHC has methodological limitations, and it is not possible to determine which side of the brain is damaged in order to cause changes in the VMHC. Furthermore, the brain structure is asymmetrical, and we try to resolve this problem using a symmetrical template. Second, although anti-Parkinsonian drugs were discontinued for 12 h, it was still difficult to avoid the long-term effects of drugs on brain function. Third, Given the metal electrodes implanted in the subjects’ brains, 1.5T MRI instead of 3.0T MRI scanner was used in this study. With the development of technology, the development of higher field strength compatible electrodes will further promote the study of deep brain stimulation mechanisms. In addition, in order to reduce patients’ head movement or discomfort caused by long collection time, the collection time of fMRI was only 128 time points. The short collection time was a disadvantage of the design of this study. In the following study, we will extend the collection time to avoid that the short collection time may affect the results of this study. Finally, the sample size of our study remains small, and more samples need to be included in the future in order to further verify our results.

## Conclusion

Overall, we found that PD patients showed decreased interhemispheric RSFC in the prefrontal cortex, cerebellum, supramarginal gyrus, and DMN-related brain regions after STN-DBS. This result indicates a disorder of hemispheric coordination after DBS. Furthermore, the positive correlation between altered VMHC value of SFG and MFG and the changed phonemic VF scores observed suggests a potential clinical implication of VMHC measure for decreased postoperative VF in PD patients. All findings provide novel insights into the pathogenesis of VF decline after DBS from an interhemispheric perspective.

## Data Availability Statement

The original contributions presented in the study are included in the article/Supplementary Material, further inquiries can be directed to the corresponding authors.

## Ethics Statement

The studies involving human participants were reviewed and approved by the Ethics Committee of Brain Hospital Affiliated with Nanjing Medical University. The patients/participants provided their written informed consent to participate in this study.

## Author Contributions

BL, WD, and CQ designed and wrote this manuscript. LC and YL collected the data. CX and DL were responsible for data processing and analysis. LZ and WL contributed to the design of the study. WZ and JY edited and revised the manuscript. All authors contributed to and approved the final manuscript.

## Conflict of Interest

The authors declare that the research was conducted in the absence of any commercial or financial relationships that could be construed as a potential conflict of interest.

## Publisher’s Note

All claims expressed in this article are solely those of the authors and do not necessarily represent those of their affiliated organizations, or those of the publisher, the editors and the reviewers. Any product that may be evaluated in this article, or claim that may be made by its manufacturer, is not guaranteed or endorsed by the publisher.

## Footnotes

1
http://rfmri.org/dpabi

2
https://www.mathworks.com/products/matlab

3
https://www.fil.ion.ucl.ac.uk/spm/software/spm12/

4
http://www.restfmri.net/

## References

[B1] Alexander M. P. Gillingham S. Schweizer T. Stuss D. T. (2012). Cognitive impairments due to focal cerebellar injuries in adults. *Cortex* 48 980–990. 10.1016/j.cortex.2011.03.012 21549360

[B2] Baldo J. V. Shimamura A. P. (1998). Letter and category fluency in patients with frontal lobe lesions. *Neuropsychology* 12 259–267. 10.1037//0894-4105.12.2.259 9556772

[B3] Baldo J. V. Shimamura A. P. Delis D. C. Kramer J. Kaplan E. (2001). Verbal and design fluency in patients with frontal lobe lesions. *J. Int. Neuropsychol. Soc* 7 586–596. 10.1017/s1355617701755063 11459110

[B4] Benabid A. L. Chabardes S. Mitrofanis J. Pollak P. (2009). Deep brain stimulation of the subthalamic nucleus for the treatment of Parkinson’s disease. *Lancet Neurol.* 8 67–81. 10.1016/s1474-4422(08)70291-6 19081516

[B5] Birn R. M. Kenworthy L. Case L. Caravella R. Jones T. B. Bandettini P. A. (2010). Neural systems supporting lexical search guided by letter and semantic category cues: a self-paced overt response fMRI study of verbal fluency. *Neuroimage* 49 1099–1107. 10.1016/j.neuroimage.2009.07.036 19632335 PMC2832834

[B6] Borden A. Wallon D. Lefaucheur R. Derrey S. Fetter D. Verin M. (2014). Does early verbal fluency decline after STN implantation predict long-term cognitive outcome after STN-DBS in Parkinson’s disease? *J. Neurol. Sci.* 346 299–302. 10.1016/j.jns.2014.07.063 25125047

[B7] Cao X. Wang X. Xue C. Zhang S. Huang Q. Liu W. (2020). A radiomics approach to predicting Parkinson’s disease by incorporating whole-brain functional activity and gray matter structure. *Front. Neurosci.* 14:751. 10.3389/fnins.2020.00751 32760248 PMC7373781

[B8] Costafreda S. G. Fu C. H. Lee L. Everitt B. Brammer M. J. David A. S. (2006). A systematic review and quantitative appraisal of fMRI studies of verbal fluency: role of the left inferior frontal gyrus. *Hum. Brain. Mapp.* 27 799–810. 10.1002/hbm.20221 16511886 PMC6871344

[B9] Costentin G. Derrey S. Gérardin E. Cruypeninck Y. Pressat-Laffouilhere T. Anouar Y. (2019). White matter tracts lesions and decline of verbal fluency after deep brain stimulation in Parkinson’s disease. *Hum. Brain Mapp.* 40 2561–2570. 10.1002/hbm.24544 30779251 PMC6865750

[B10] De Smet H. J. Paquier P. Verhoeven J. Mariën P. (2013). The cerebellum: its role in language and related cognitive and affective functions. *Brain Lang.* 127 334–342. 10.1016/j.bandl.2012.11.001 23333152

[B11] Deuschl G. Schade-Brittinger C. Krack P. Volkmann J. Schäfer H. Bötzel K. (2006). A randomized trial of deep-brain stimulation for Parkinson’s disease. *N. Engl. J. Med.* 355 896–908. 10.1056/NEJMoa060281 16943402

[B12] Dimond S. J. (1979). Performance by split-brain humans on lateralized vigilance tasks. *Cortex* 15 43–50. 10.1016/s0010-9452(79)80005-2 446044

[B13] Ding X. Li C. Y. Wang Q. S. Du F. Z. Ke Z. W. Peng F. (2014). Patterns in default-mode network connectivity for determining outcomes in cognitive function in acute stroke patients. *Neuroscience* 277 637–646. 10.1016/j.neuroscience.2014.07.060 25090922

[B14] Dubois B. Burn D. Goetz C. Aarsland D. Brown R. G. Broe G. A. (2007). Diagnostic procedures for Parkinson’s disease dementia: recommendations from the movement disorder society task force. *Mov. Disord.* 22 2314–2324. 10.1002/mds.21844 18098298

[B15] Gan C. Wang L. Ji M. Ma K. Sun H. Zhang K. (2021). Abnormal interhemispheric resting state functional connectivity in Parkinson’s disease patients with impulse control disorders. *NPJ Parkinsons Dis.* 7:60. 10.1038/s41531-021-00205-7 34272398 PMC8285494

[B16] Gan C. Wang M. Si Q. Yuan Y. Zhi Y. Wang L. (2020). Altered interhemispheric synchrony in Parkinson’s disease patients with levodopa-induced dyskinesias. *NPJ Parkinsons Dis.* 6:14. 10.1038/s41531-020-0116-2 32665973 PMC7343784

[B17] Geva S. Schneider L. M. Roberts S. Green D. W. Price C. J. (2021). The effect of focal damage to the right medial posterior cerebellum on word and sentence comprehension and production. *Front. Hum. Neurosci.* 15:664650. 10.3389/fnhum.2021.664650 34093152 PMC8172582

[B18] Grogan A. Parker Jones O. Ali N. Crinion J. Orabona S. Mechias M. L. (2012). Structural correlates for lexical efficiency and number of languages in non-native speakers of english. *Neuropsychologia* 50 1347–1352. 10.1016/j.neuropsychologia.2012.02.019 22401989 PMC3382713

[B19] Guo W. Xiao C. Liu G. Wooderson S. C. Zhang Z. Zhang J. (2014). Decreased resting-state interhemispheric coordination in first-episode, drug-naive paranoid schizophrenia. *Prog. Neuropsychopharmacol. Biol. Psychiatry* 48 14–19. 10.1016/j.pnpbp.2013.09.012 24075897

[B20] Henry J. D. Crawford J. R. (2004). Verbal fluency deficits in Parkinson’s disease: a meta-analysis. *J. Int. Neuropsychol. Soc.* 10 608–622. 10.1017/s1355617704104141 15327739

[B21] Hu X. Zhang J. Jiang X. Zhou C. Wei L. Yin X. (2015). Decreased interhemispheric functional connectivity in subtypes of Parkinson’s disease. *J. Neurol.* 262 760–767. 10.1007/s00415-014-7627-x 25577177

[B22] Hubrich-Ungureanu P. Kaemmerer N. Henn F. A. Braus D. F. (2002). Lateralized organization of the cerebellum in a silent verbal fluency task: a functional magnetic resonance imaging study in healthy volunteers. *Neurosci. Lett.* 319 91–94. 10.1016/s0304-3940(01)02566-6 11825678

[B23] Janowsky J. S. Shimamura A. P. Kritchevsky M. Squire L. R. (1989). Cognitive impairment following frontal lobe damage and its relevance to human amnesia. *Behav. Neurosci.* 103 548–560. 10.1037//0735-7044.103.3.548 2736069

[B24] Jin C. Qi S. Teng Y. Li C. Yao Y. Ruan X. (2021). Integrating structural and functional interhemispheric brain connectivity of gait freezing in Parkinson’s disease. *Front. Neurol.* 12:609866. 10.3389/fneur.2021.609866 33935931 PMC8081966

[B25] Le Goff F. Derrey S. Lefaucheur R. Borden A. Fetter D. Jan M. (2015). Decline in verbal fluency after subthalamic nucleus deep brain stimulation in Parkinson’s disease: a microlesion effect of the electrode trajectory? *J. Parkinsons Dis.* 5 95–104. 10.3233/jpd-140443 25374271

[B26] Lee H. Devlin J. T. Shakeshaft C. Stewart L. H. Brennan A. Glensman J. (2007). Anatomical traces of vocabulary acquisition in the adolescent brain. *J. Neurosci.* 27 1184–1189. 10.1523/jneurosci.4442-06.2007 17267574 PMC6673201

[B27] Lefaucheur R. Derrey S. Martinaud O. Wallon D. Chastan N. Gérardin E. (2012). Early verbal fluency decline after STN implantation: is it a cognitive microlesion effect? *J. Neurol. Sci.* 321 96–99. 10.1016/j.jns.2012.07.033 22846795

[B28] Li J. Yuan Y. Wang M. Zhang J. Zhang L. Jiang S. (2018). Decreased interhemispheric homotopic connectivity in Parkinson’s disease patients with freezing of gait: a resting state fMRI study. *Parkinsonism Relat. Disord.* 52 30–36. 10.1016/j.parkreldis.2018.03.015 29602542

[B29] Liao J. Li T. Dong W. Wang J. Tian J. Liu J. (2019). Reduced prefrontal-temporal cortical activation during verbal fluency task in obsessive-compulsive disorder: a multi-channel near-infrared spectroscopy study. *J. Psychiatr. Res.* 109 33–40. 10.1016/j.jpsychires.2018.11.006 30468975

[B30] Liao Z. L. Tan Y. F. Qiu Y. J. Zhu J. P. Chen Y. Lin S. S. (2018). Interhemispheric functional connectivity for Alzheimer’s disease and amnestic mild cognitive impairment based on the triple network model. *J. Zhejiang Univ. Sci. B* 19 924–934. 10.1631/jzus.B1800381 30507076 PMC6305256

[B31] Luo C. Guo X. Song W. Zhao B. Cao B. Yang J. (2015). Decreased resting-state interhemispheric functional connectivity in Parkinson’s disease. *Biomed. Res. Int.* 2015:692684. 10.1155/2015/692684 26180807 PMC4477209

[B32] Mikos A. Bowers D. Noecker A. M. McIntyre C. C. Won M. Chaturvedi A. (2011). Patient-specific analysis of the relationship between the volume of tissue activated during DBS and verbal fluency. *Neuroimage* 54 S238–S246. 10.1016/j.neuroimage.2010.03.068 20362061 PMC2908727

[B33] Morrison C. E. Borod J. C. Perrine K. Beric A. Brin M. F. Rezai A. (2004). Neuropsychological functioning following bilateral subthalamic nucleus stimulation in Parkinson’s disease. *Arch. Clin. Neuropsychol.* 19 165–181. 10.1016/s0887-6177(03)00004-0 15010083

[B34] Pereira J. B. Junqué C. Martí M. J. Ramirez-Ruiz B. Bartrés-Faz D. Tolosa E. (2009). Structural brain correlates of verbal fluency in Parkinson’s disease. *Neuroreport* 20 741–744. 10.1097/WNR.0b013e328329370b 19349926

[B35] Pozzilli C. Bastianello S. Padovani A. Passafiume D. Millefiorini E. Bozzao L. (1991). Anterior corpus callosum atrophy and verbal fluency in multiple sclerosis. *Cortex* 27 441–445. 10.1016/s0010-9452(13)80039-1 1743039

[B36] Quan W. Wu T. Li Z. Wang Y. Dong W. Lv B. (2015). Reduced prefrontal activation during a verbal fluency task in Chinese-speaking patients with schizophrenia as measured by near-infrared spectroscopy. *Prog. Neuropsychopharmacol. Biol. Psychiatry* 58 51–58. 10.1016/j.pnpbp.2014.12.005 25542372

[B37] Sauerwein H. C. Lassonde M. (1994). Cognitive and sensori-motor functioning in the absence of the corpus callosum: neuropsychological studies in callosal agenesis and callosotomized patients. *Behav. Brain Res.* 64 229–240. 10.1016/0166-4328(94)90135-x 7840889

[B38] Smitha K. A. Arun K. M. Rajesh P. G. Thomas B. Radhakrishnan A. Sarma P. S. (2019). Resting fMRI as an alternative for task-based fMRI for language lateralization in temporal lobe epilepsy patients: a study using independent component analysis. *Neuroradiology* 61 803–810. 10.1007/s00234-019-02209-w 31020344

[B39] Starowicz-Filip A. Chrobak A. A. Moskała M. Krzyżewski R. M. Kwinta B. Kwiatkowski S. (2017). The role of the cerebellum in the regulation of language functions. *Psychiatr. Pol.* 51 661–671. 10.12740/pp/68547 28987056

[B40] Thompson-Schill S. L. Swick D. Farah M. J. D’Esposito M. Kan I. P. Knight R. T. (1998). Verb generation in patients with focal frontal lesions: a neuropsychological test of neuroimaging findings. *Proc. Natl. Acad. Sci. U S A* 95 15855–15860. 10.1073/pnas.95.26.15855 9861060 PMC28134

[B41] Vitali P. Abutalebi J. Tettamanti M. Rowe J. Scifo P. Fazio F. (2005). Generating animal and tool names: an fMRI study of effective connectivity. *Brain Lang.* 93 32–45. 10.1016/j.bandl.2004.08.005 15766766

[B42] Witt K. Daniels C. Reiff J. Krack P. Volkmann J. Pinsker M. O. (2008). Neuropsychological and psychiatric changes after deep brain stimulation for Parkinson’s disease: a randomised, multicentre study. *Lancet. Neurol.* 7 605–614. 10.1016/s1474-4422(08)70114-5 18538636

[B43] Witt K. Pulkowski U. Herzog J. Lorenz D. Hamel W. Deuschl G. (2004). Deep brain stimulation of the subthalamic nucleus improves cognitive flexibility but impairs response inhibition in Parkinso’n disease. *Arch. Neurol.* 61 697–700. 10.1001/archneur.61.5.697 15148146

[B44] Wolters A. F. van de Weijer S. C. F. Leentjens A. F. G. Duits A. A. Jacobs H. I. L. Kuijf M. L. (2019). Resting-state fMRI in Parkinson’s disease patients with cognitive impairment: a meta-analysis. *Parkinsonism Relat. Disord.* 62 16–27. 10.1016/j.parkreldis.2018.12.016 30580907

[B45] Yaldizli Ö Penner I. K. Frontzek K. Naegelin Y. Amann M. Papadopoulou A. (2014). The relationship between total and regional corpus callosum atrophy, cognitive impairment and fatigue in multiple sclerosis patients. *Mult. Scler.* 20 356–364. 10.1177/1352458513496880 23959709

[B46] Yang J. Ji X. Quan W. Liu Y. Wei B. Wu T. (2020). Classification of schizophrenia by functional connectivity strength using functional near infrared spectroscopy. *Front. Neuroinform.* 14:40. 10.3389/fninf.2020.00040 33117140 PMC7575761

[B47] Yuan P. Raz N. (2014). Prefrontal cortex and executive functions in healthy adults: a meta-analysis of structural neuroimaging studies. *Neurosci. Biobehav. Rev.* 42 180–192. 10.1016/j.neubiorev.2014.02.005 24568942 PMC4011981

[B48] Zhu Y. Song X. Xu M. Hu X. Li E. Liu J. (2016). Impaired interhemispheric synchrony in Parkinson’s disease with depression. *Sci. Rep.* 6:27477. 10.1038/srep27477 27265427 PMC4893739

[B49] Zuo X. N. Kelly C. Di Martino A. Mennes M. Margulies D. S. Bangaru S. (2010). Growing together and growing apart: regional and sex differences in the lifespan developmental trajectories of functional homotopy. *J. Neurosci.* 30 15034–15043. 10.1523/jneurosci.2612-10.2010 21068309 PMC2997358

