# Supplementary figures and images for: Altered Interhemispheric Functional Connectivity Associated With Early Verbal Fluency Decline After Deep Brain Stimulation in Parkinson’s Disease
Source: Front Aging Neurosci. 2022 Apr 1;14:799545. doi: 10.3389/fnagi.2022.799545 (PMC9011328; doi:10.3389/fnagi.2022.799545)

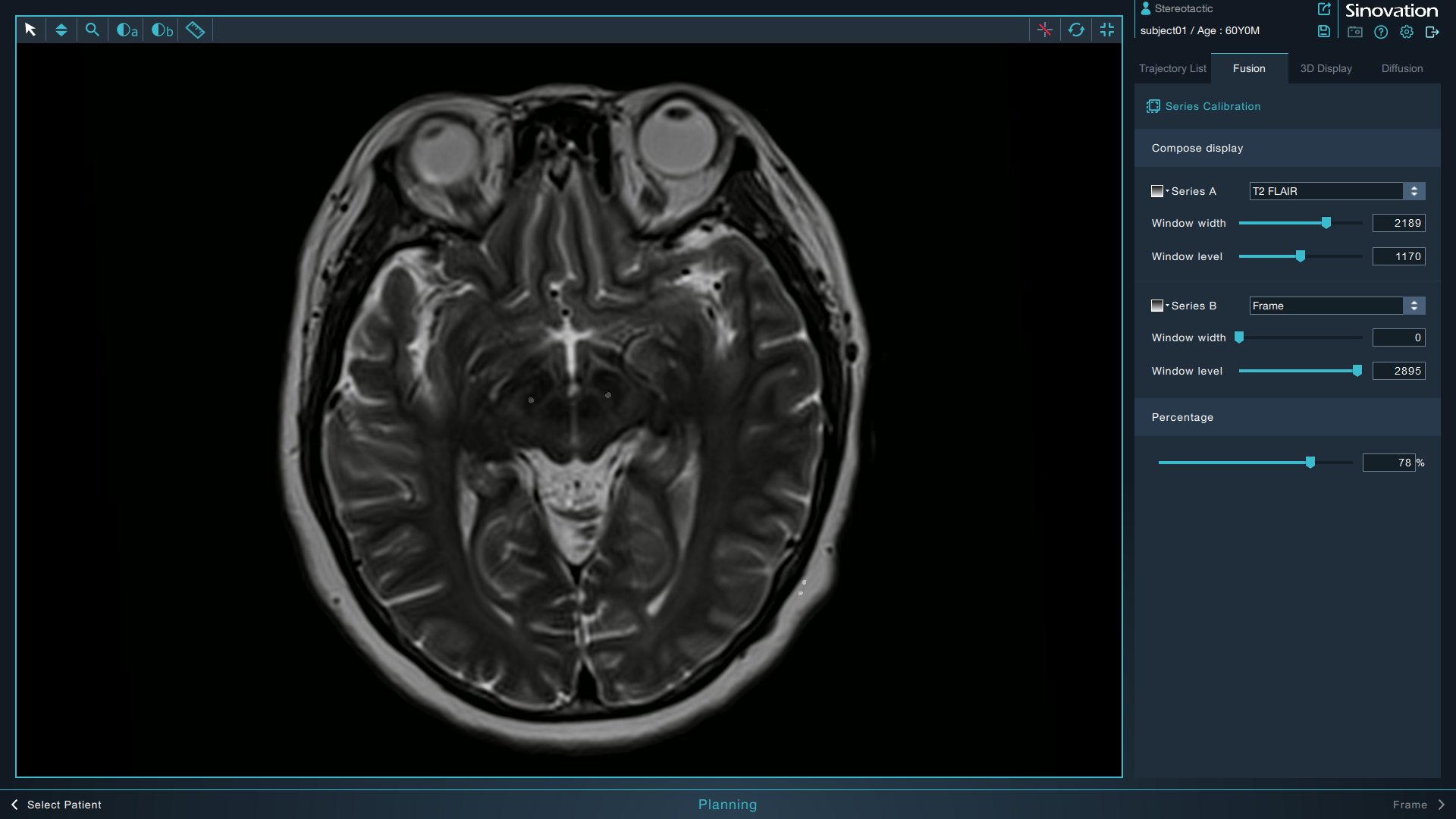

Supplement: Supplementary file 1 [file Data_Sheet_1.ZIP › Postoperative electrode position/subject01.png]

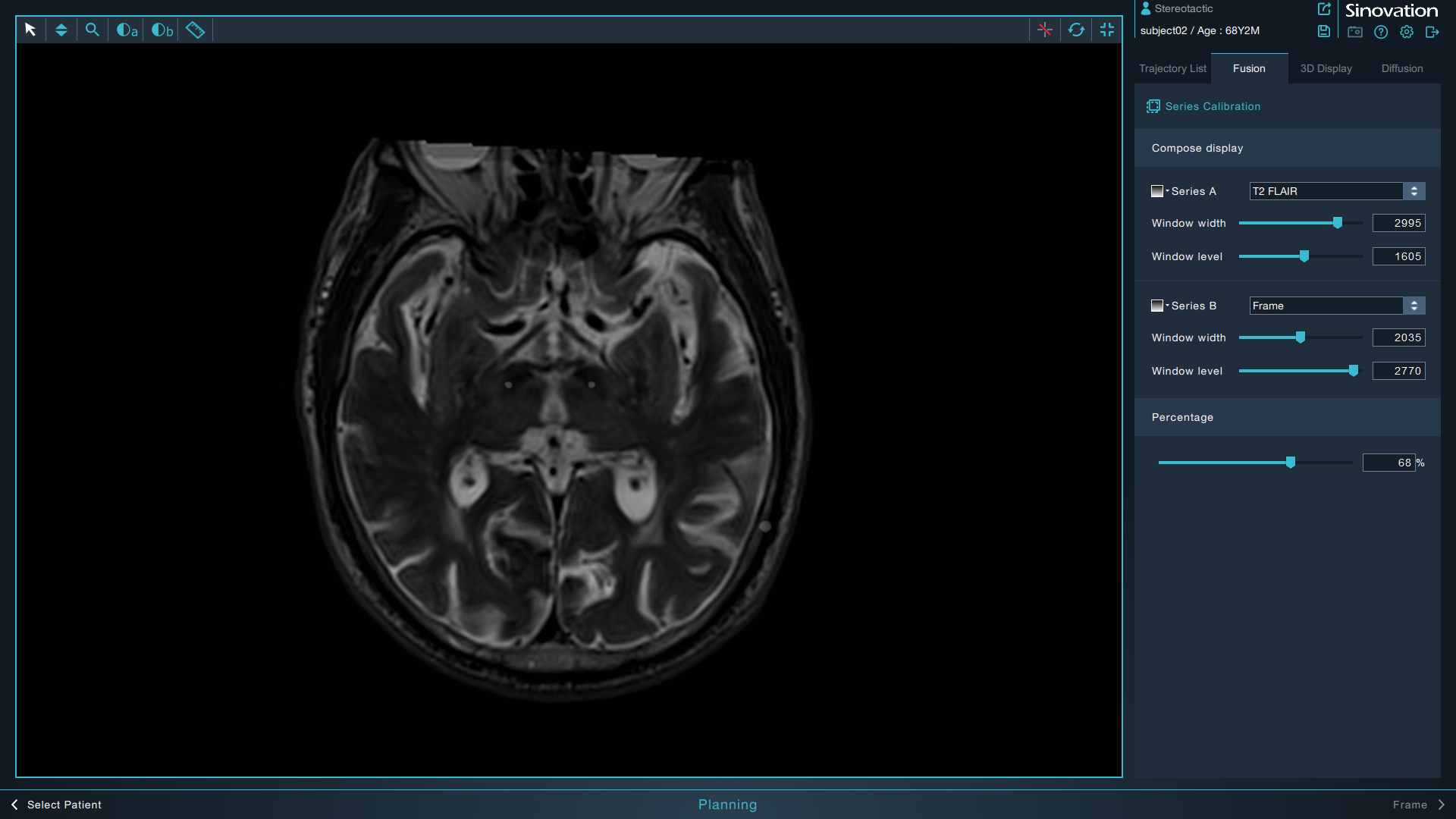

Supplement: Supplementary file 1 [file Data_Sheet_1.ZIP › Postoperative electrode position/subject02.png]

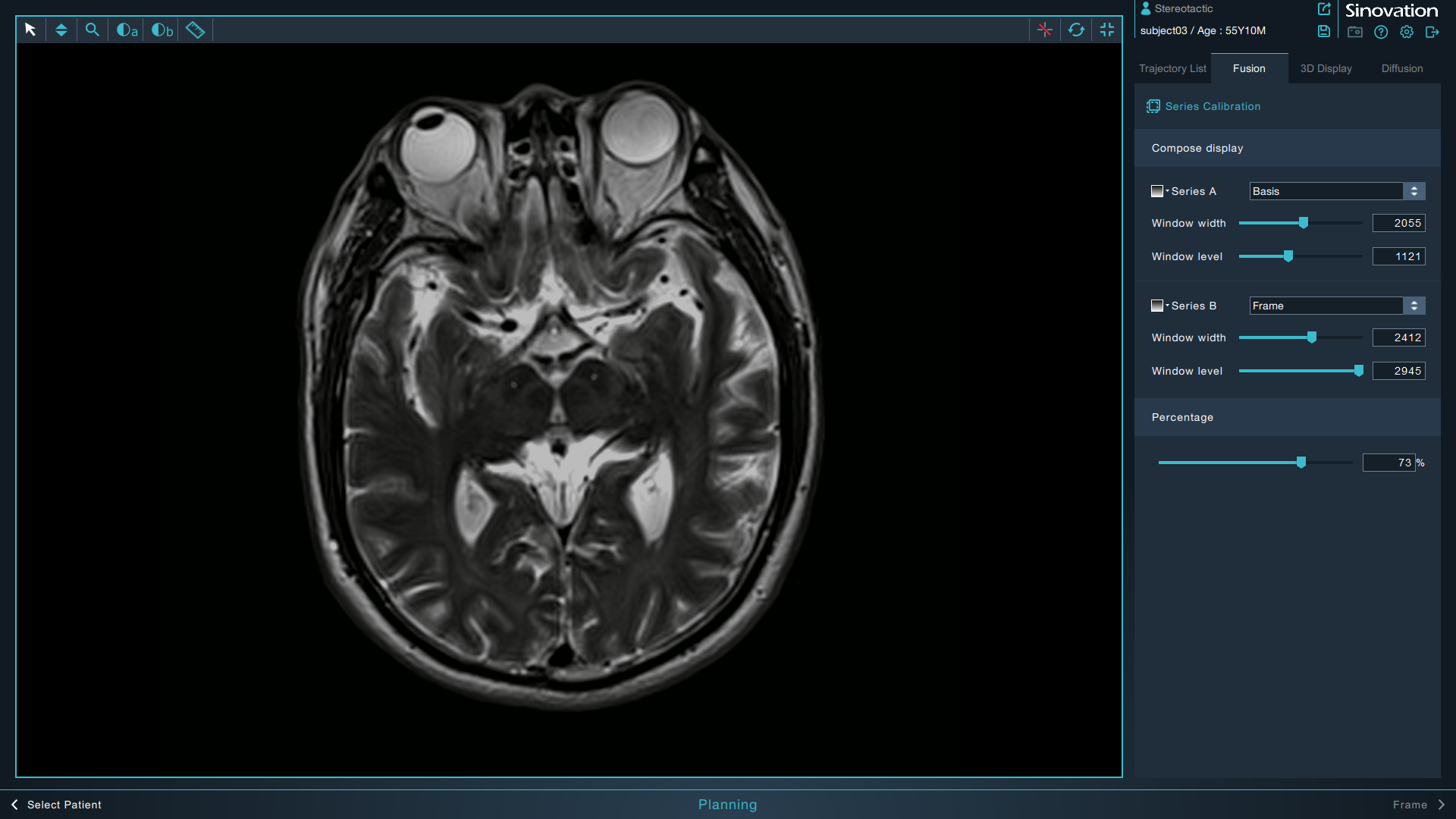

Supplement: Supplementary file 1 [file Data_Sheet_1.ZIP › Postoperative electrode position/subject03.png]

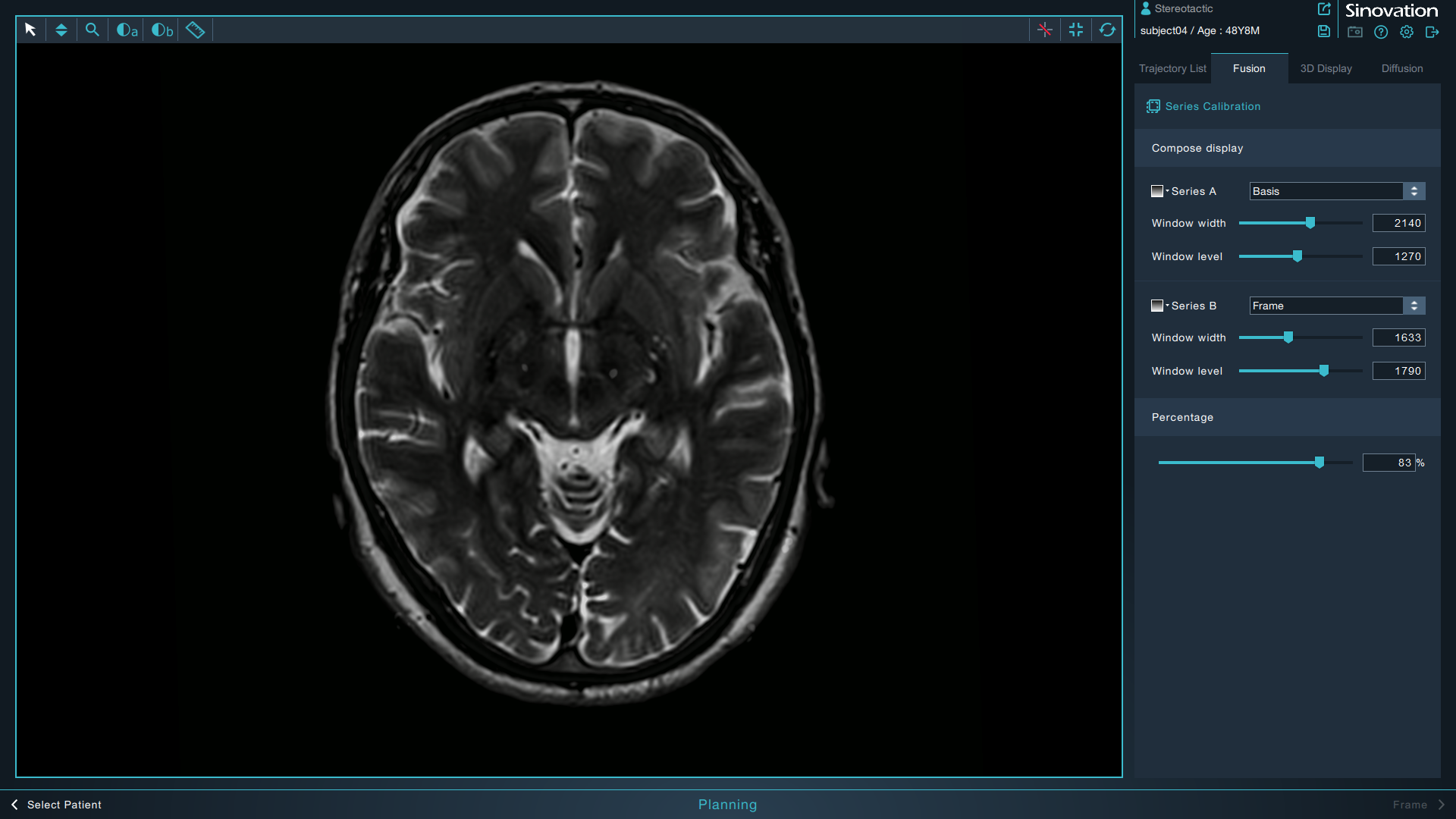

Supplement: Supplementary file 1 [file Data_Sheet_1.ZIP › Postoperative electrode position/subject04.png]

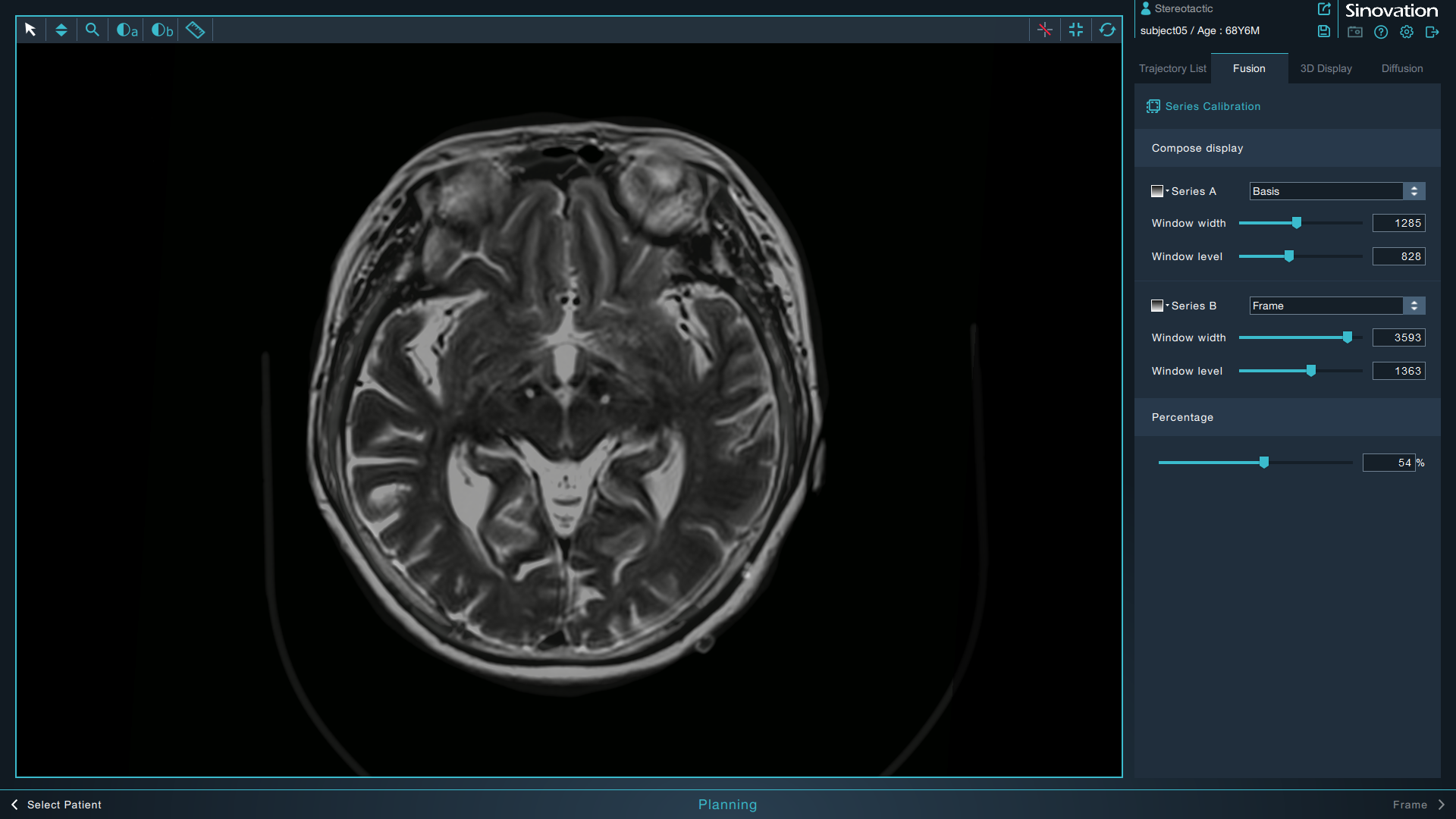

Supplement: Supplementary file 1 [file Data_Sheet_1.ZIP › Postoperative electrode position/subject05.png]

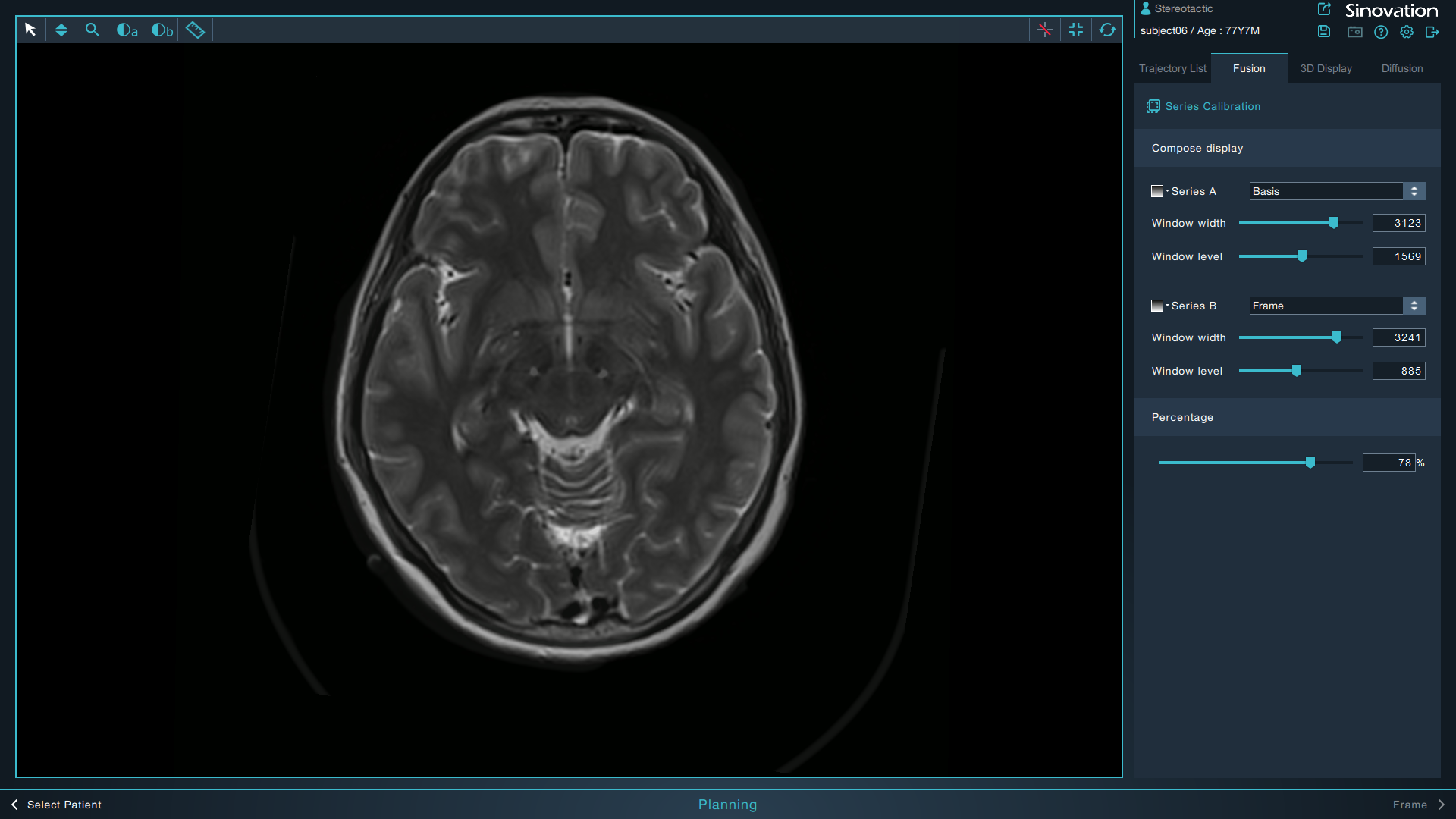

Supplement: Supplementary file 1 [file Data_Sheet_1.ZIP › Postoperative electrode position/subject06.png]

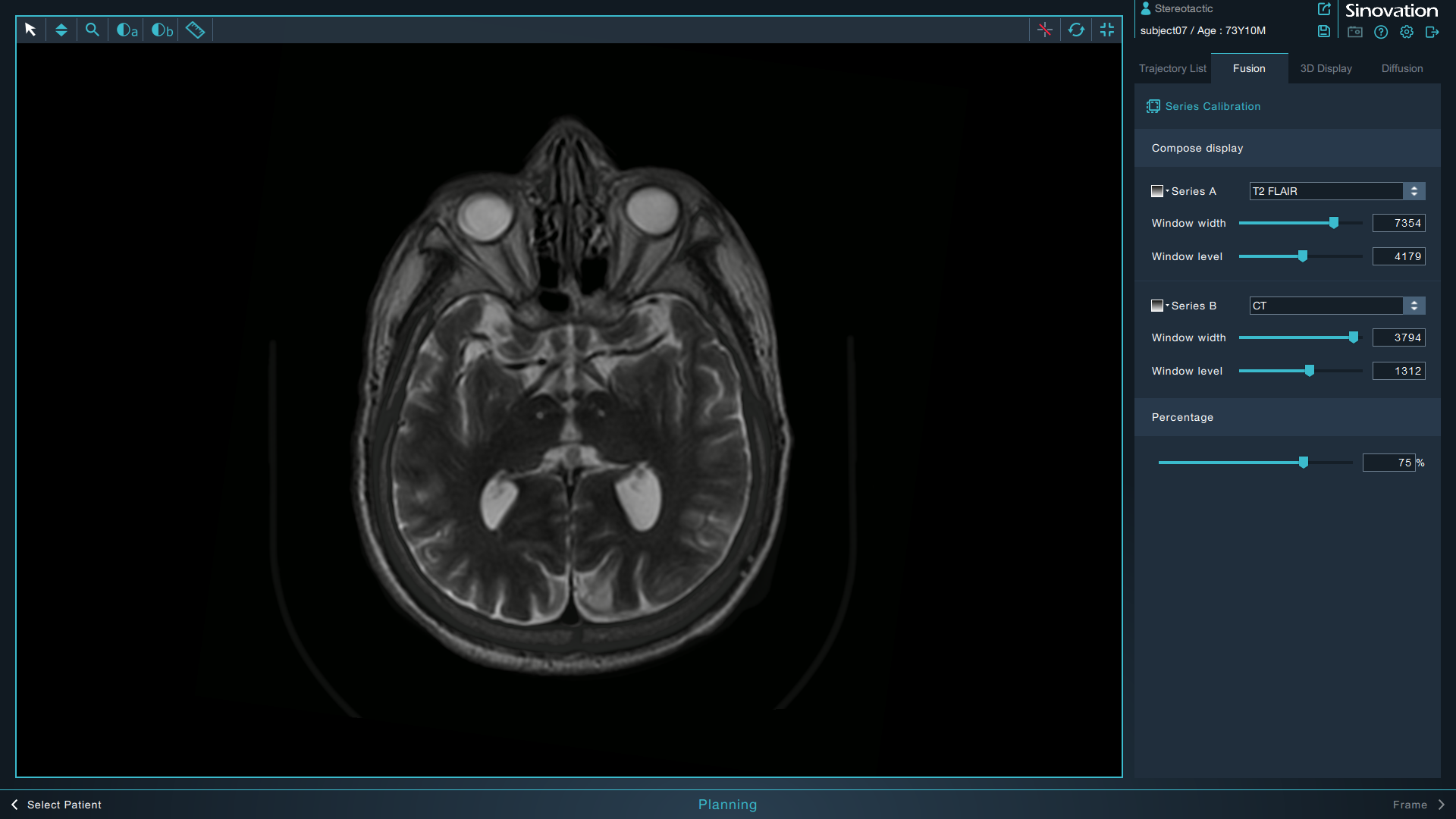

Supplement: Supplementary file 1 [file Data_Sheet_1.ZIP › Postoperative electrode position/subject07.png]

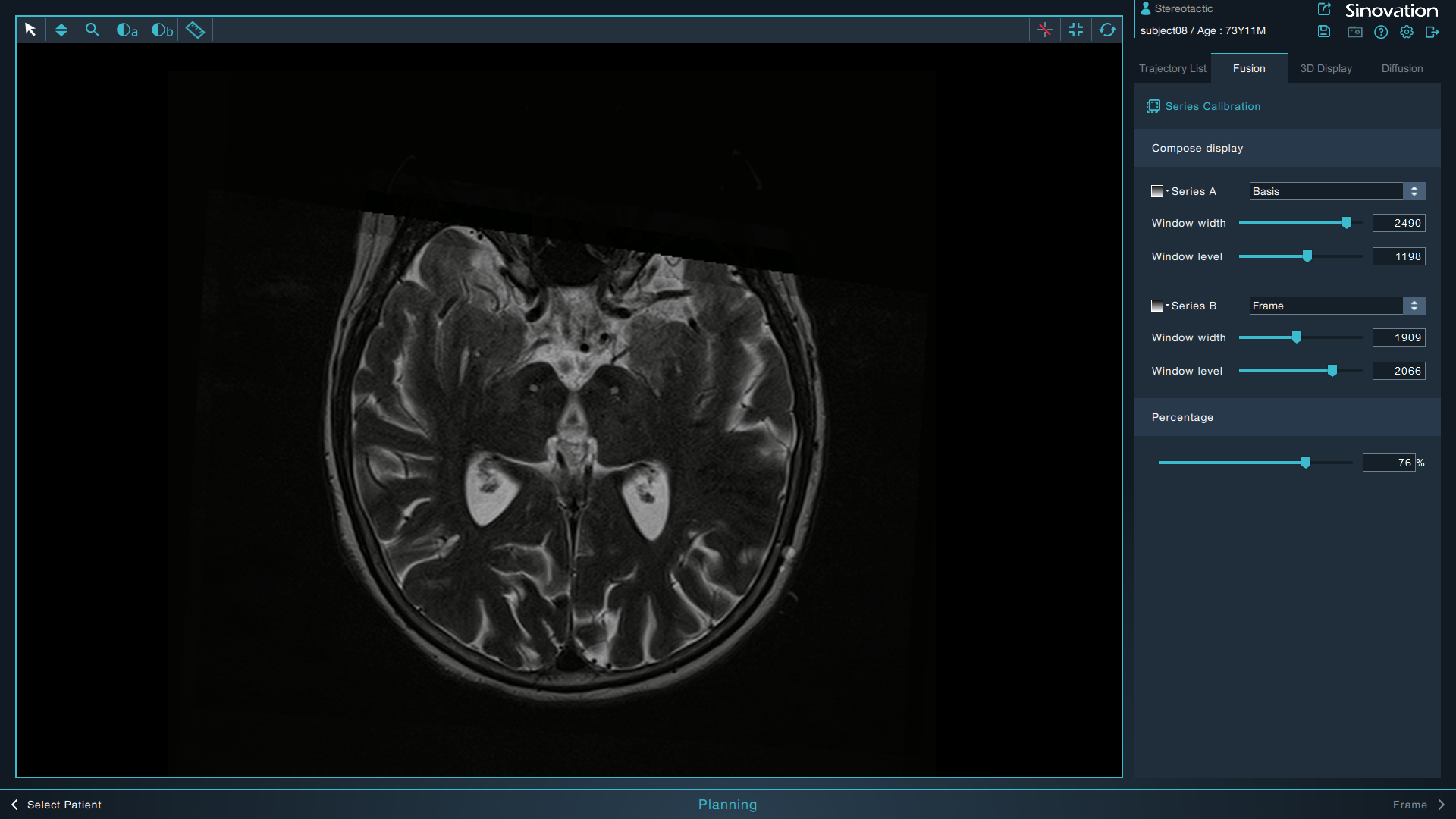

Supplement: Supplementary file 1 [file Data_Sheet_1.ZIP › Postoperative electrode position/subject08.png]

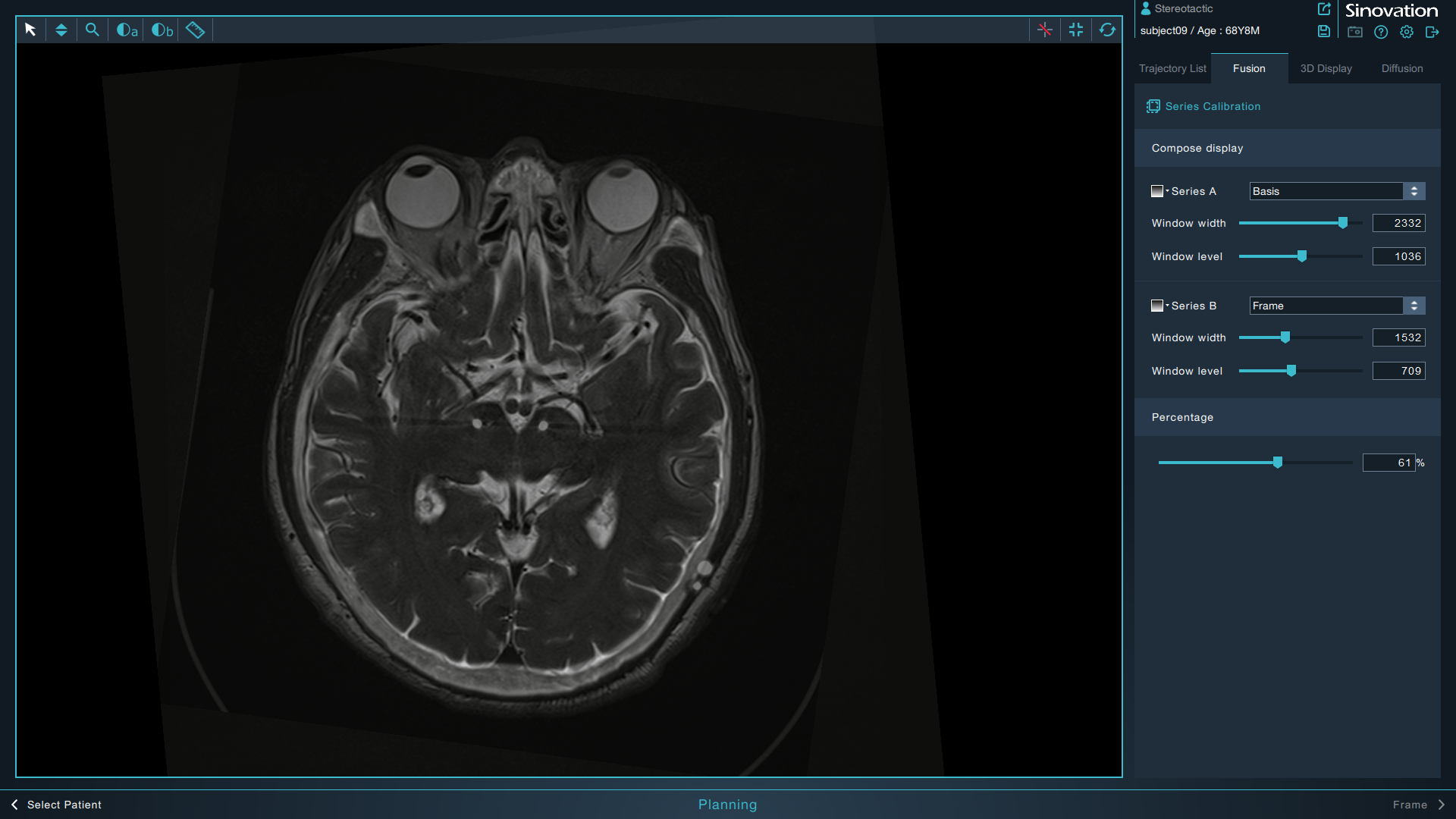

Supplement: Supplementary file 1 [file Data_Sheet_1.ZIP › Postoperative electrode position/subject09.png]

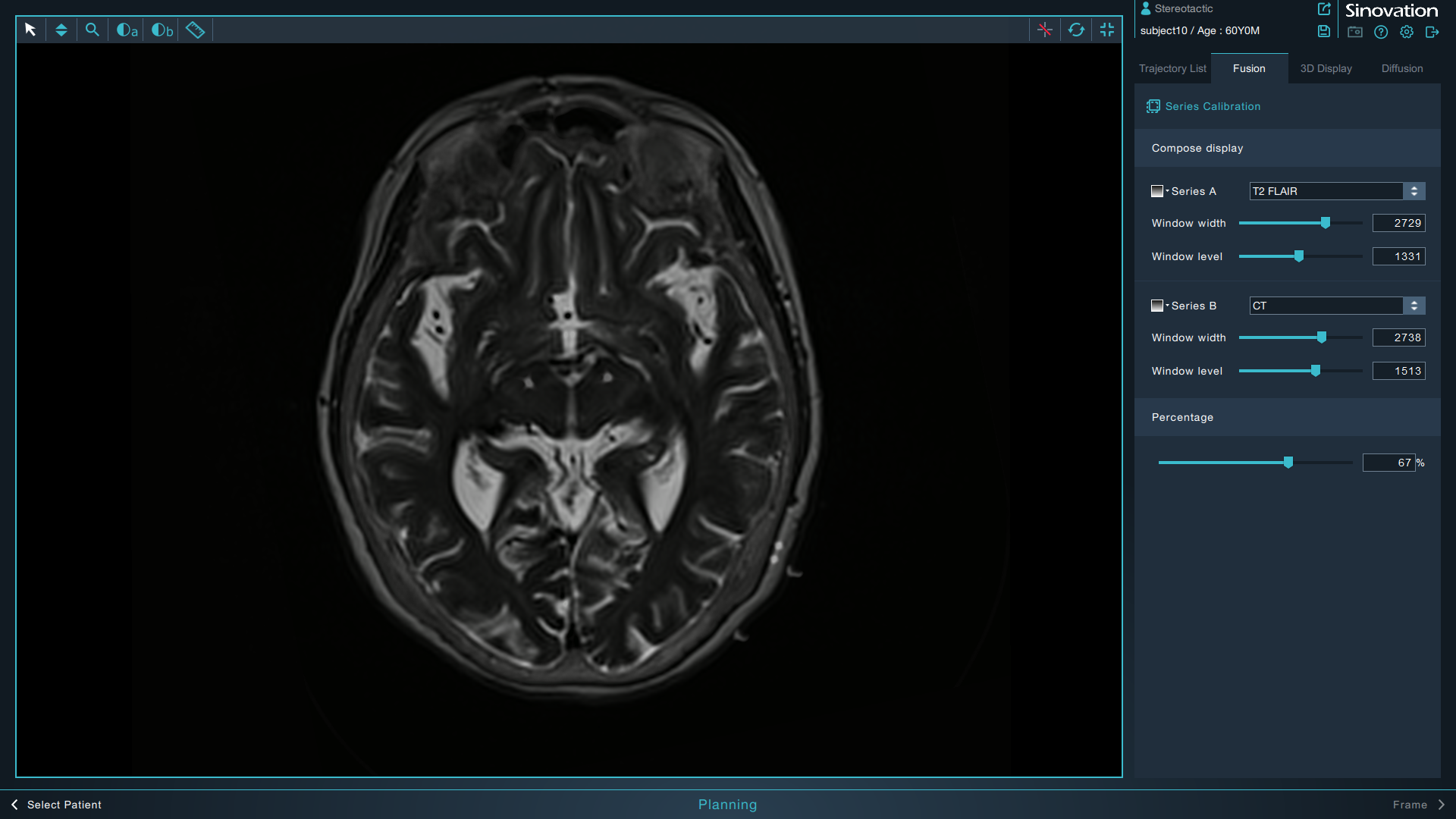

Supplement: Supplementary file 1 [file Data_Sheet_1.ZIP › Postoperative electrode position/subject10.png]

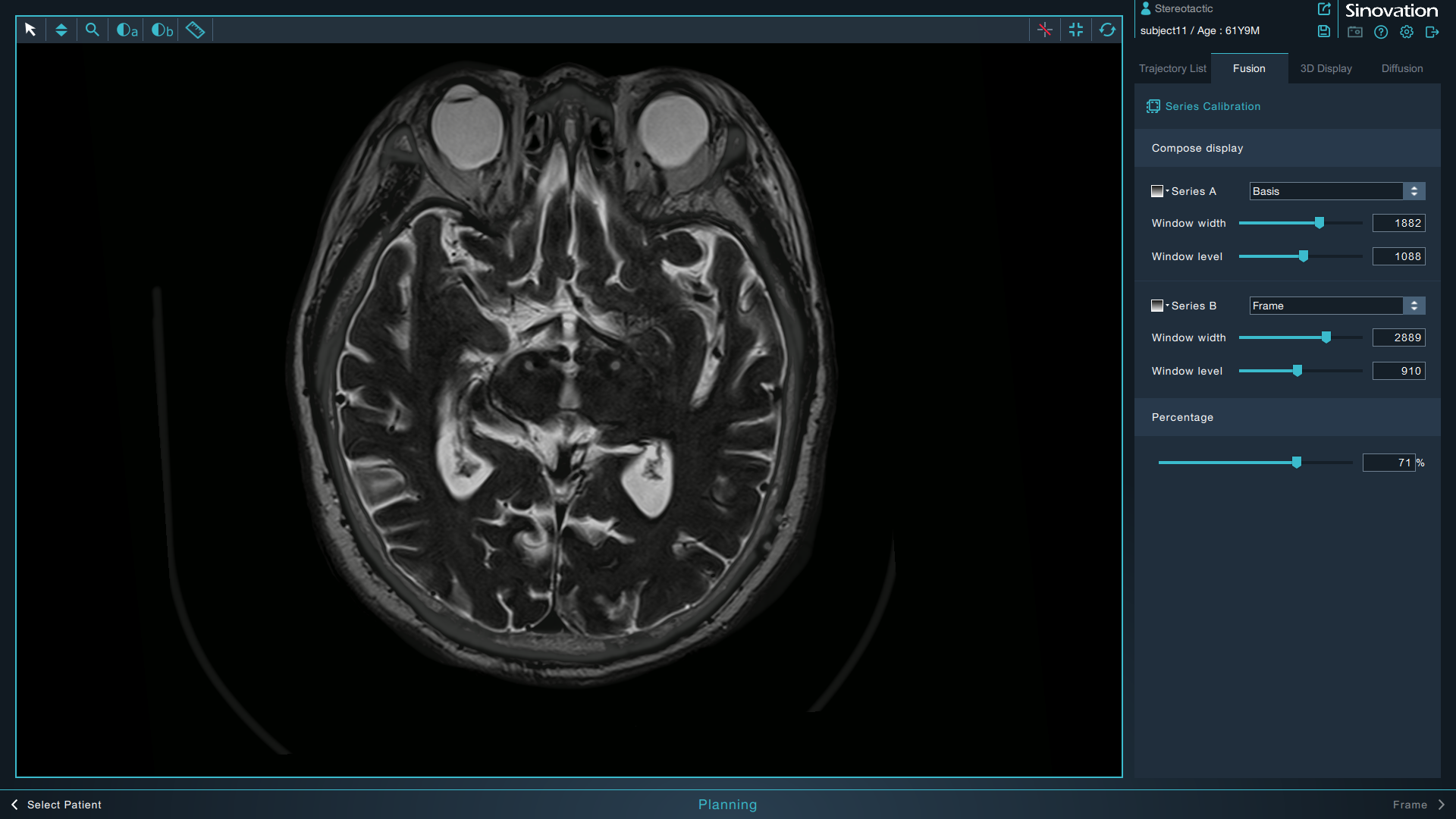

Supplement: Supplementary file 1 [file Data_Sheet_1.ZIP › Postoperative electrode position/subject11.png]

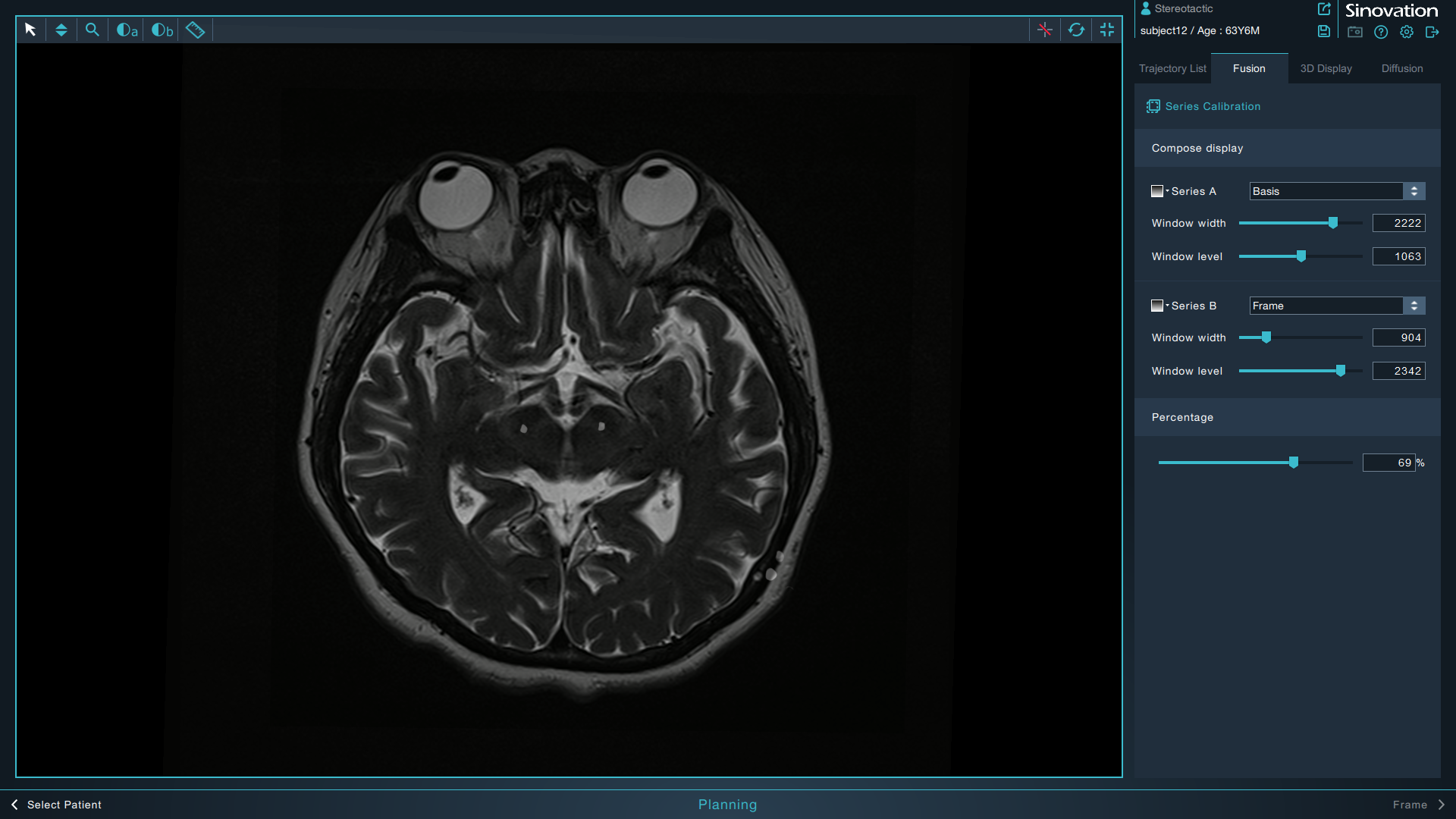

Supplement: Supplementary file 1 [file Data_Sheet_1.ZIP › Postoperative electrode position/subject12.png]

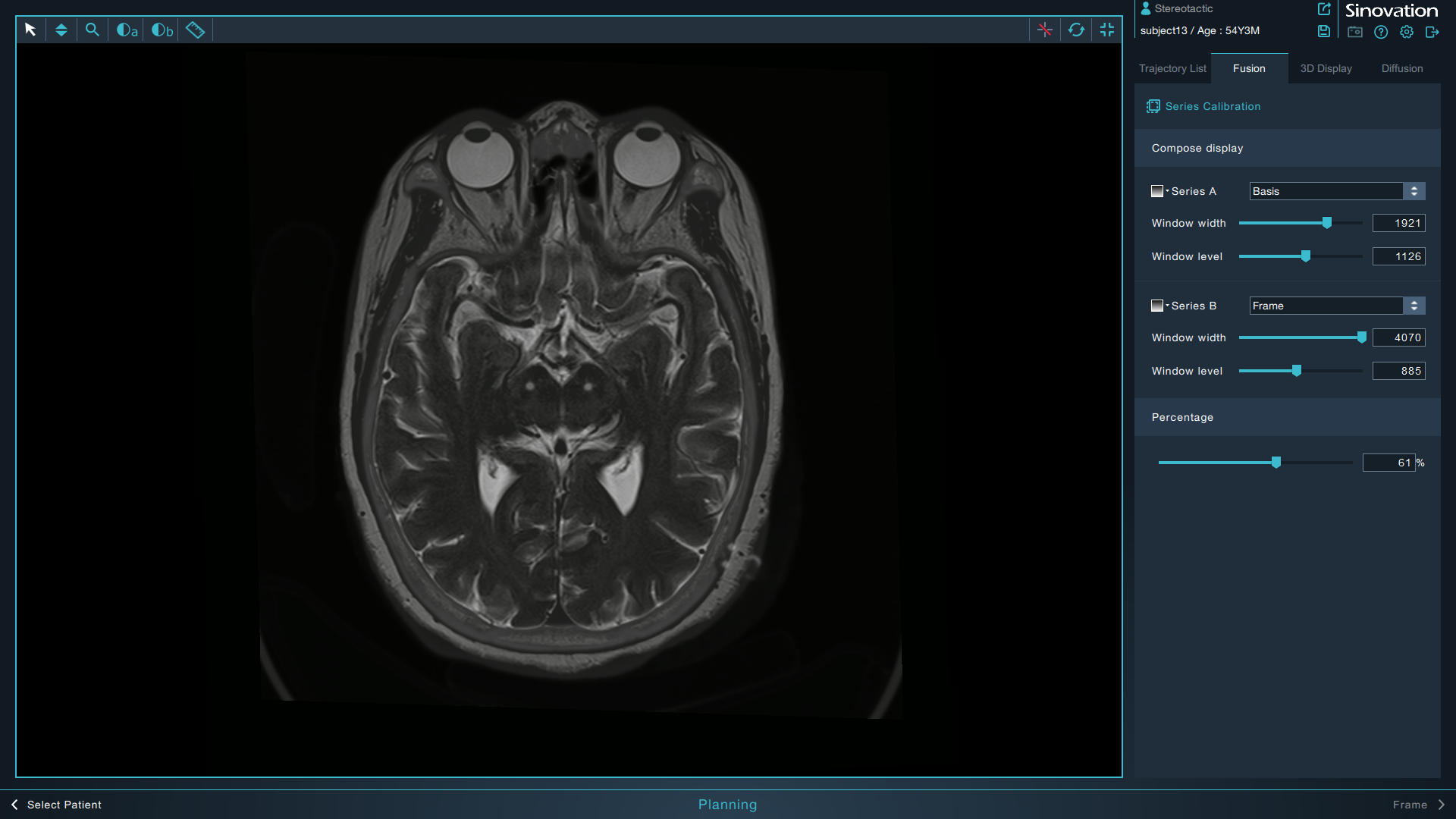

Supplement: Supplementary file 1 [file Data_Sheet_1.ZIP › Postoperative electrode position/subject13.png]

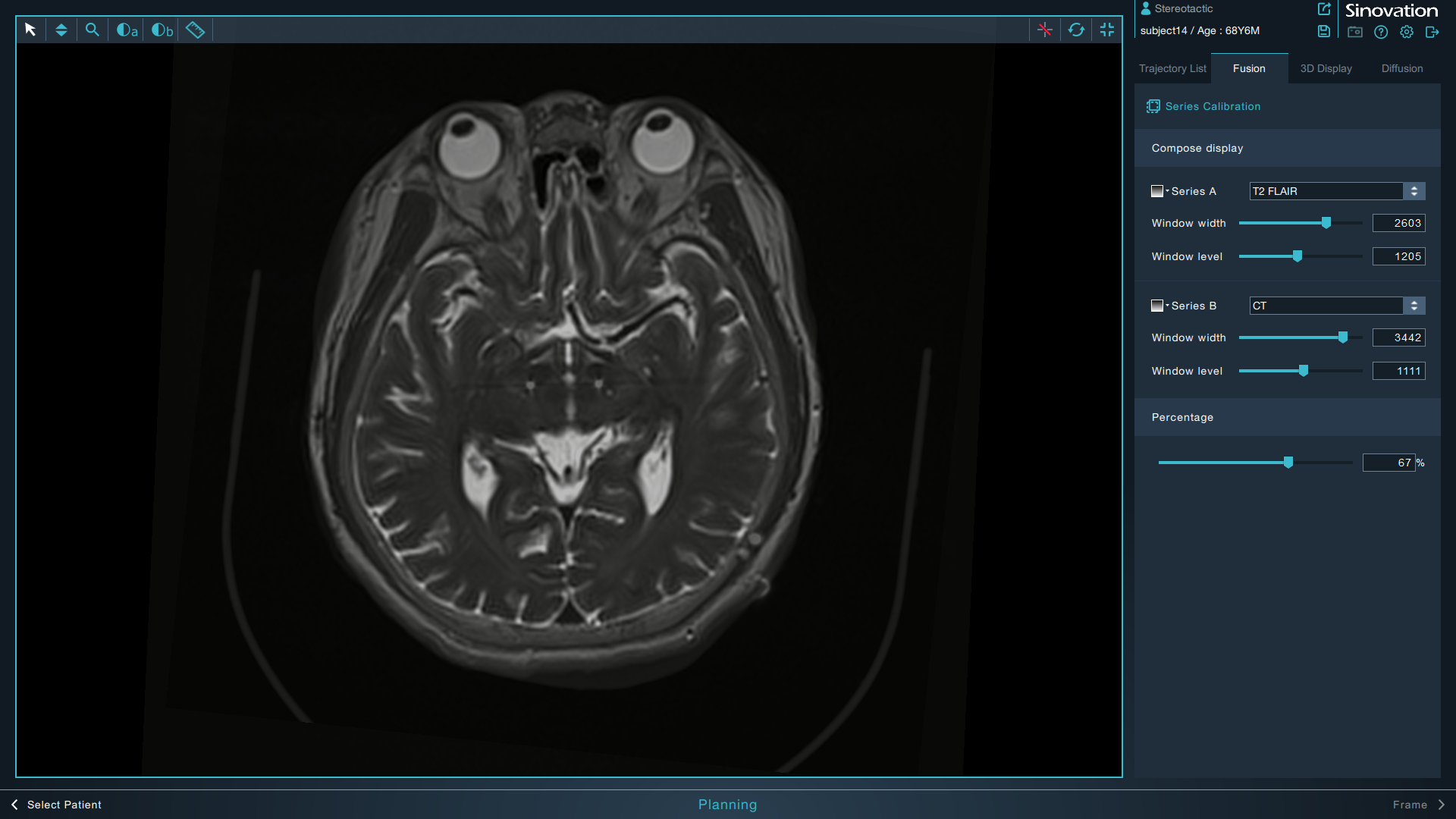

Supplement: Supplementary file 1 [file Data_Sheet_1.ZIP › Postoperative electrode position/subject14.png]

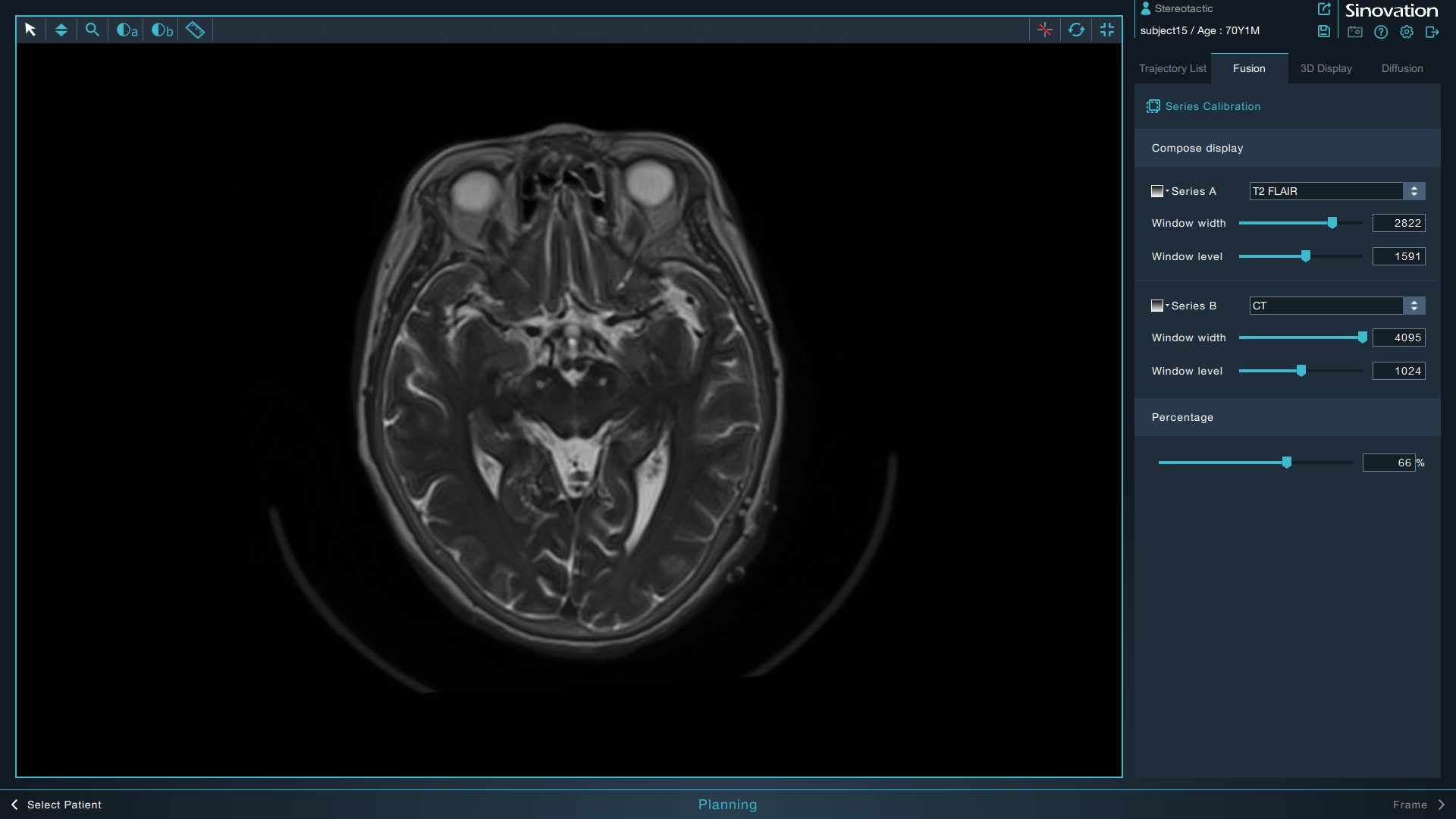

Supplement: Supplementary file 1 [file Data_Sheet_1.ZIP › Postoperative electrode position/subject15.png]

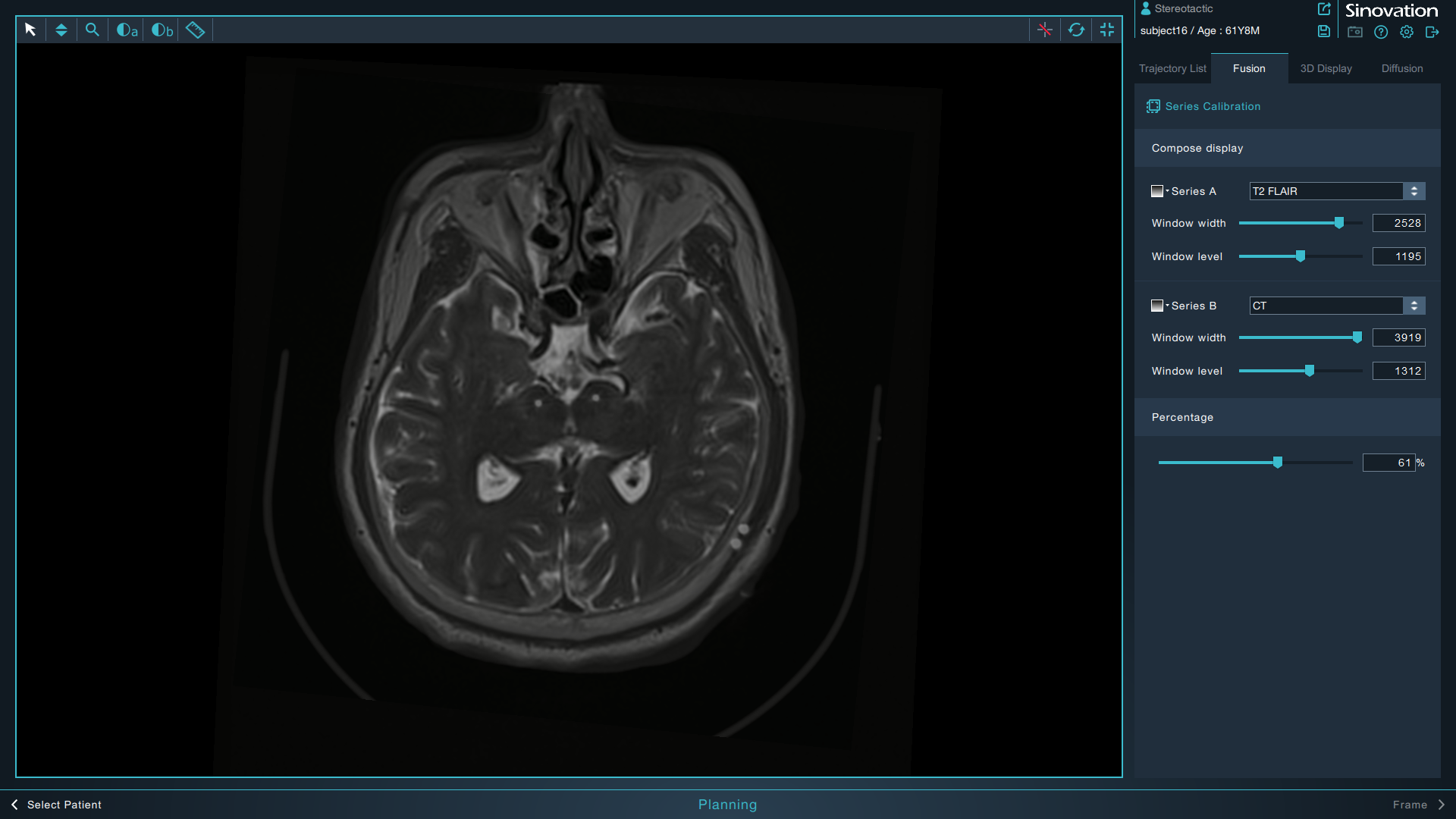

Supplement: Supplementary file 1 [file Data_Sheet_1.ZIP › Postoperative electrode position/subject16.png]

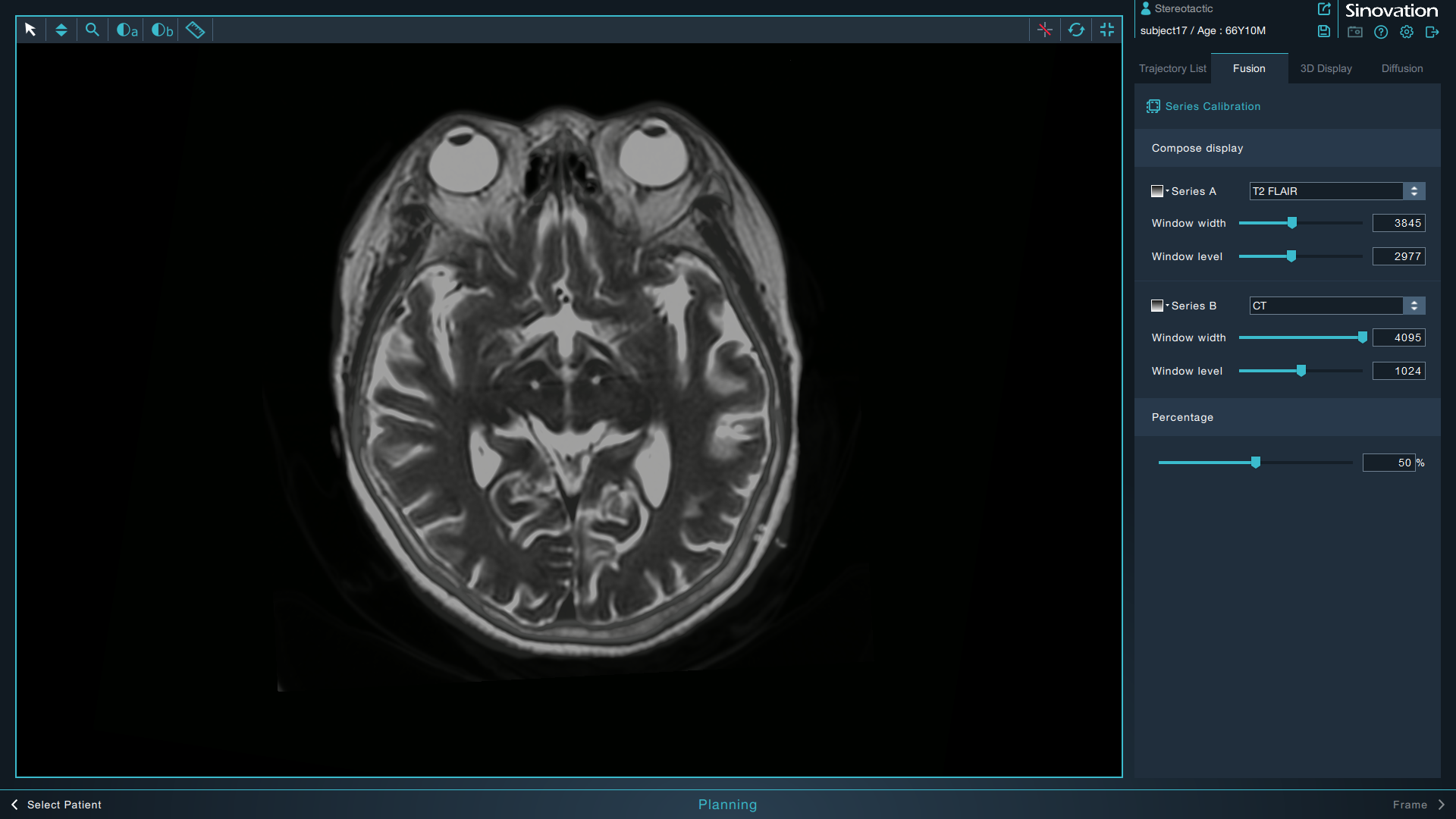

Supplement: Supplementary file 1 [file Data_Sheet_1.ZIP › Postoperative electrode position/subject17.png]

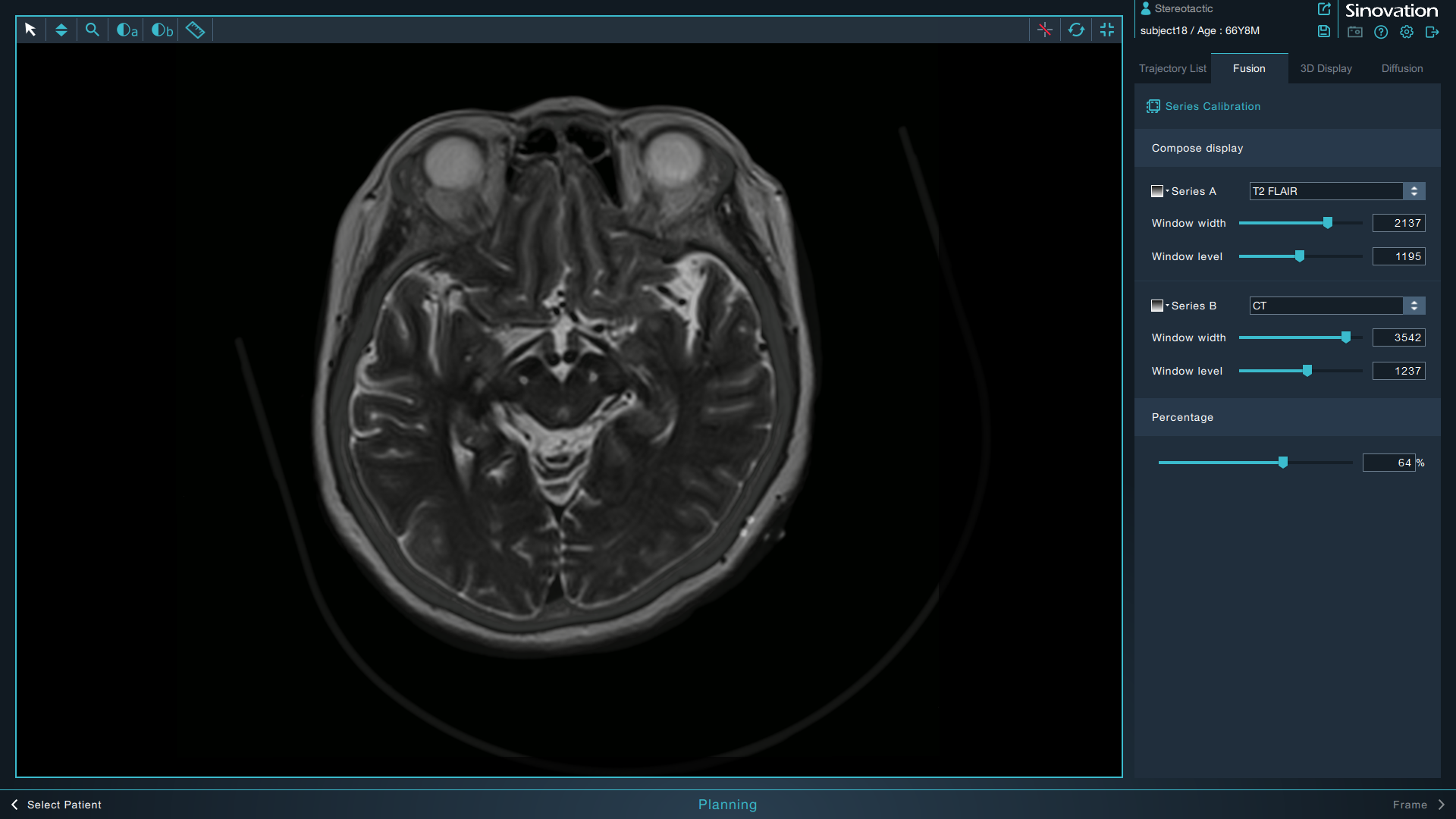

Supplement: Supplementary file 1 [file Data_Sheet_1.ZIP › Postoperative electrode position/subject18.png]

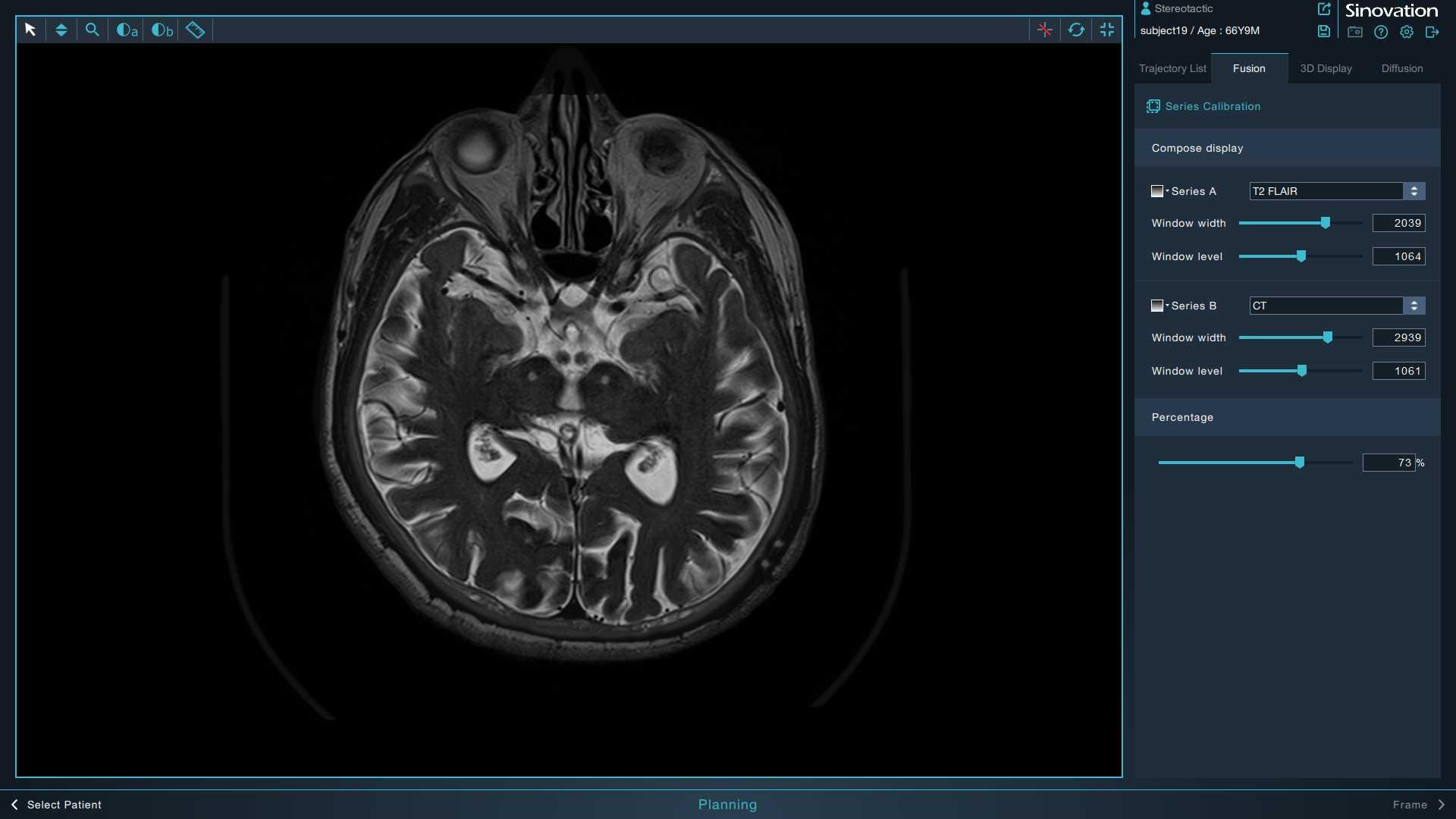

Supplement: Supplementary file 1 [file Data_Sheet_1.ZIP › Postoperative electrode position/subject19.png]

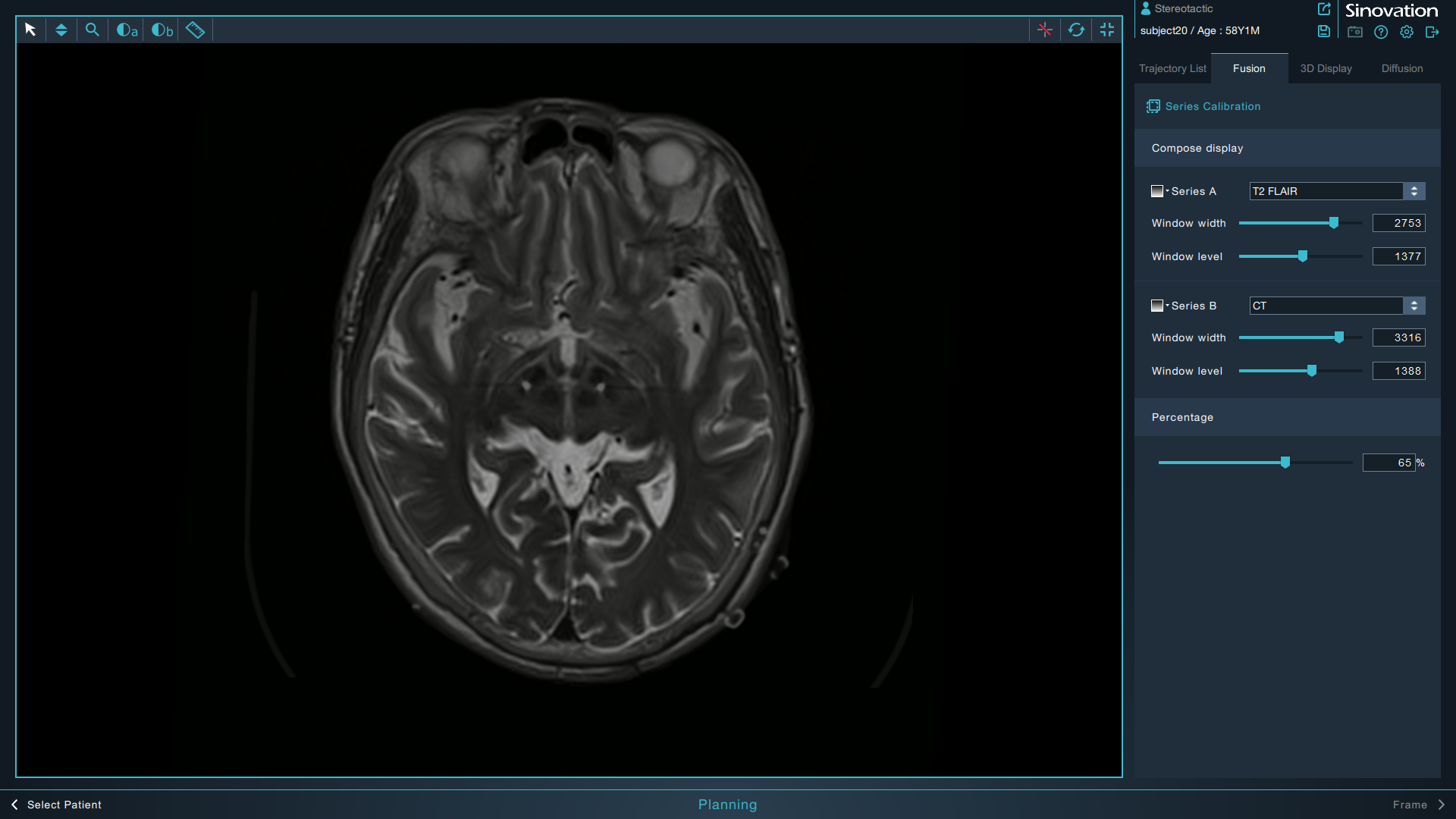

Supplement: Supplementary file 1 [file Data_Sheet_1.ZIP › Postoperative electrode position/subject20.png]

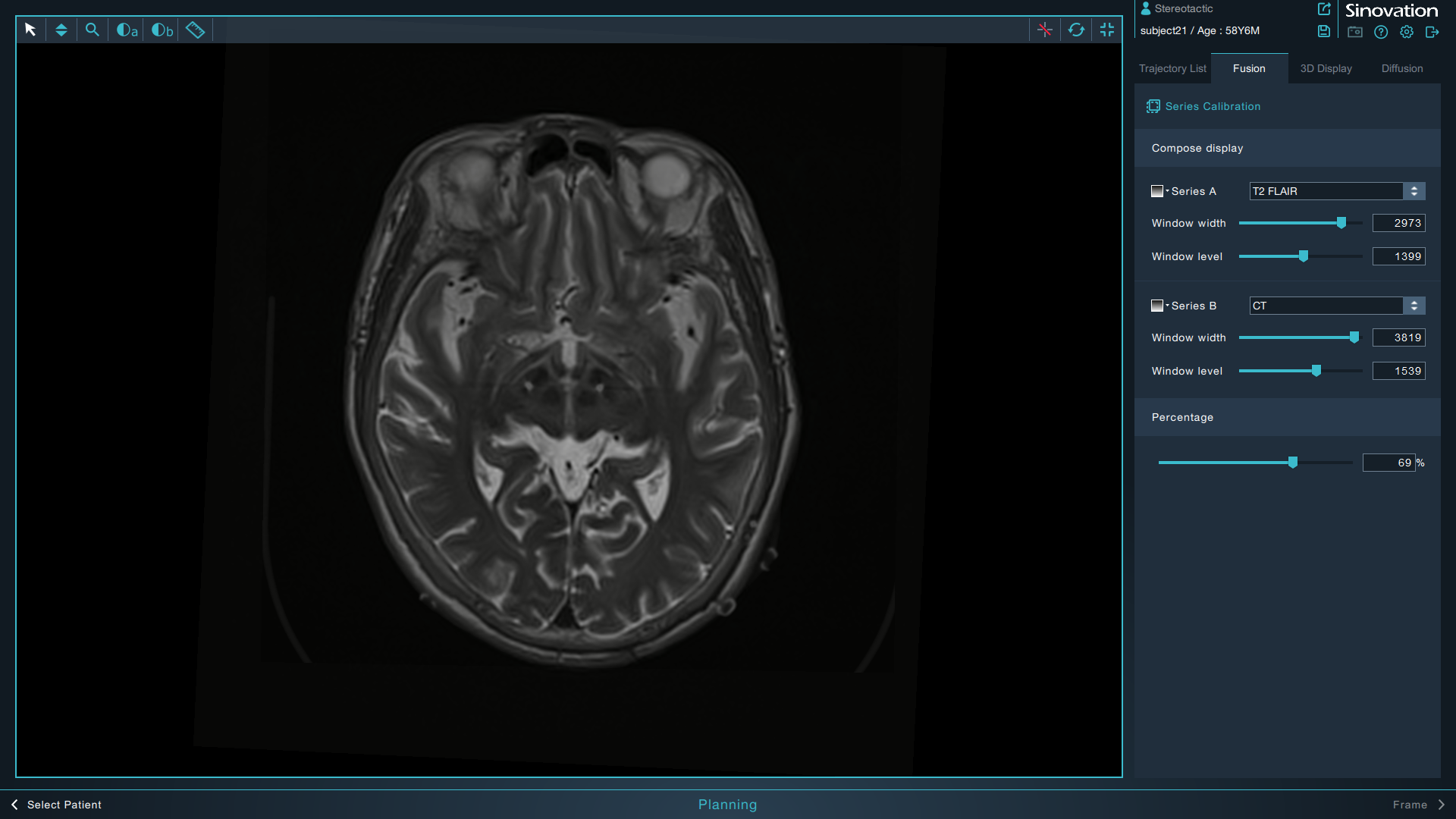

Supplement: Supplementary file 1 [file Data_Sheet_1.ZIP › Postoperative electrode position/subject21.png]

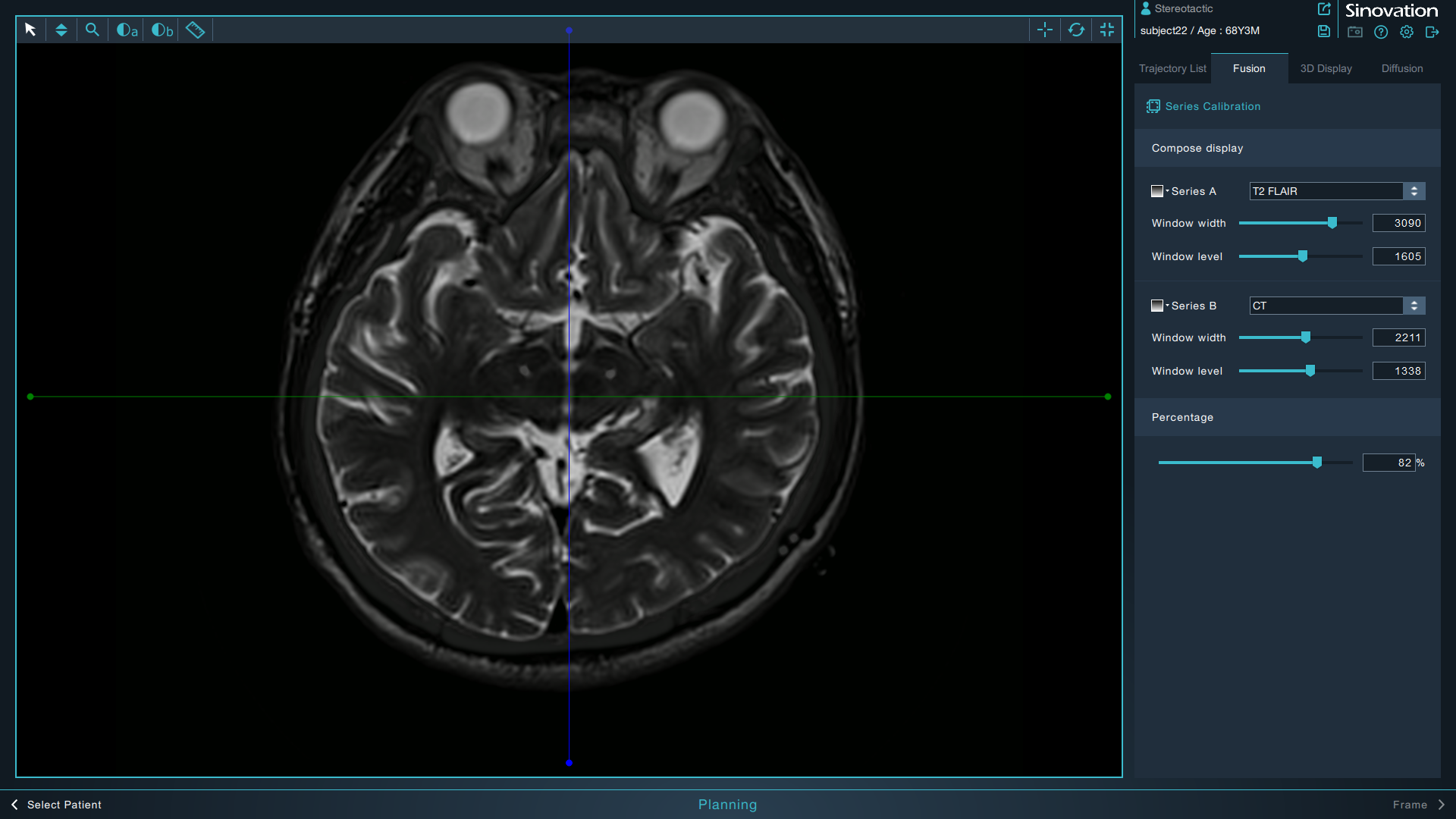

Supplement: Supplementary file 1 [file Data_Sheet_1.ZIP › Postoperative electrode position/subject22.png]

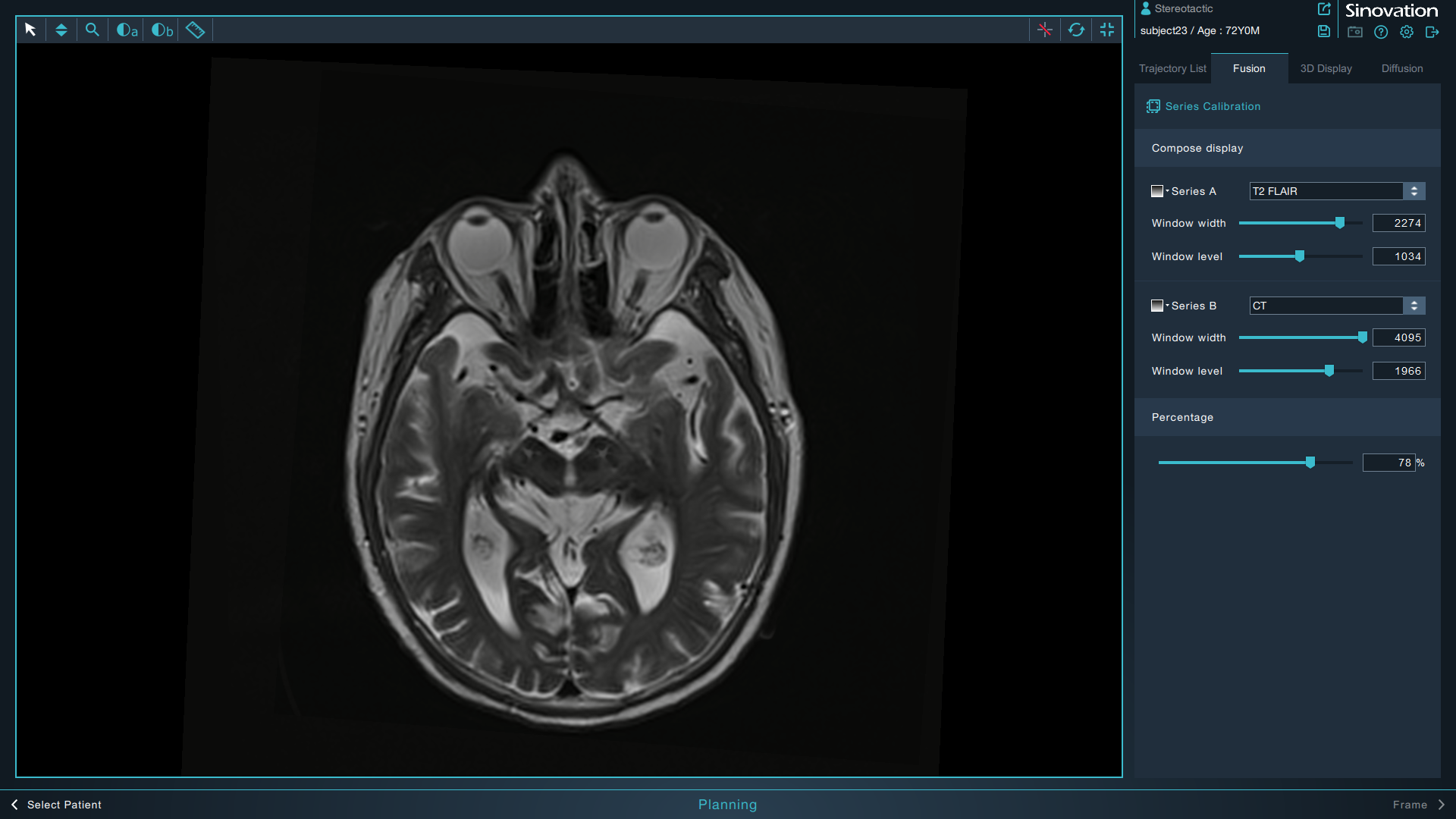

Supplement: Supplementary file 1 [file Data_Sheet_1.ZIP › Postoperative electrode position/subject23.png]

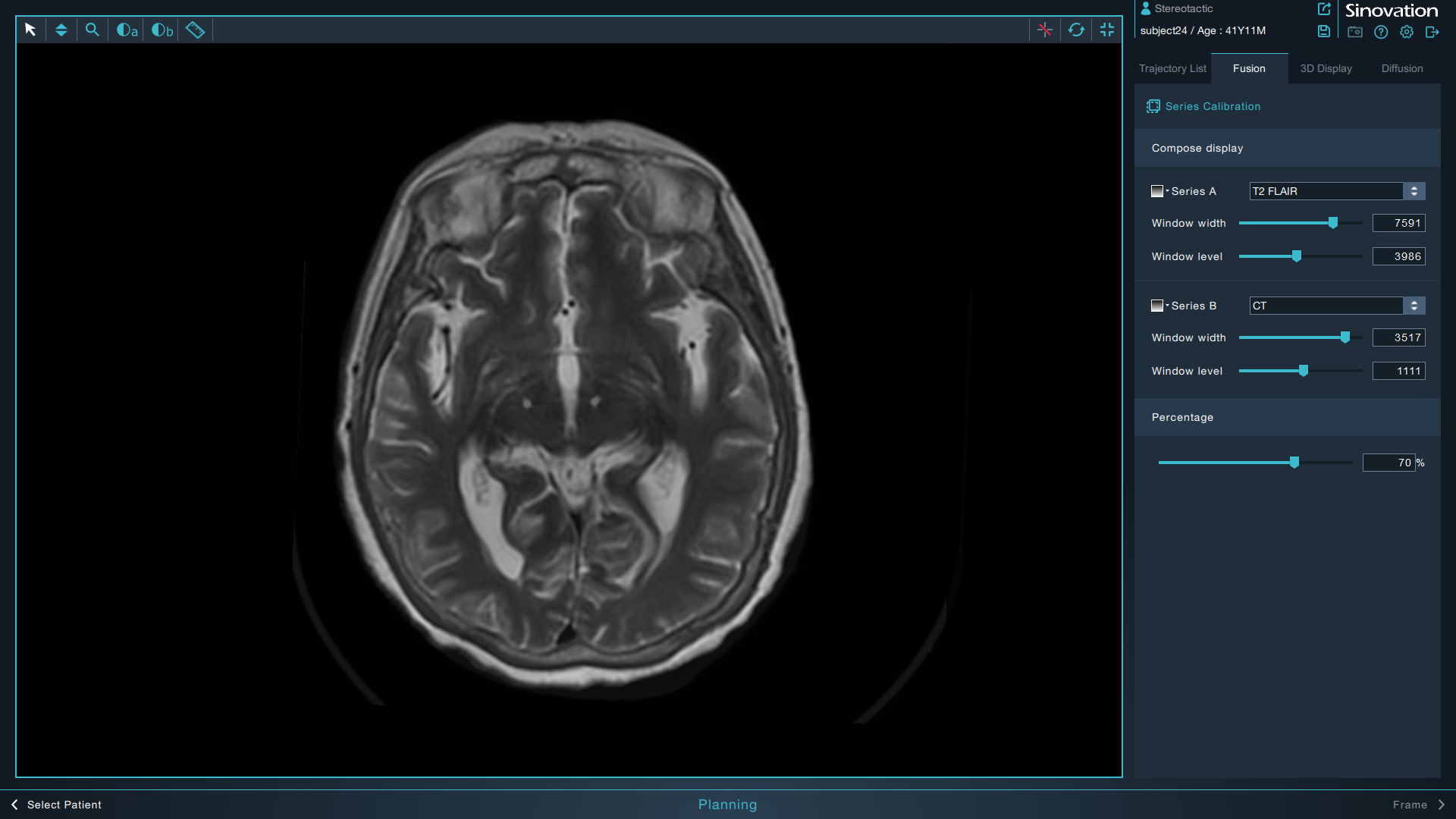

Supplement: Supplementary file 1 [file Data_Sheet_1.ZIP › Postoperative electrode position/subject24.png]

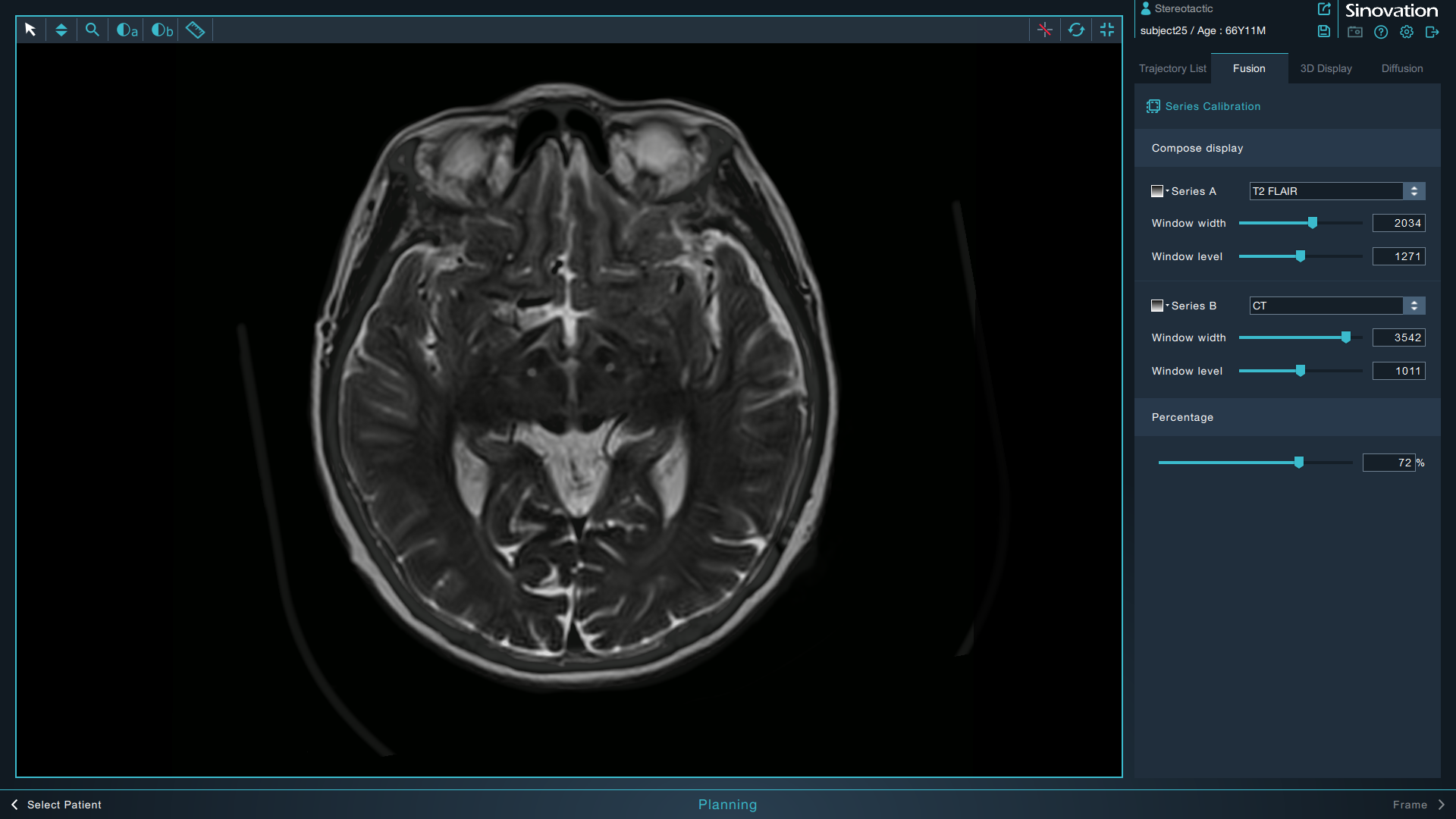

Supplement: Supplementary file 1 [file Data_Sheet_1.ZIP › Postoperative electrode position/subject25.png]

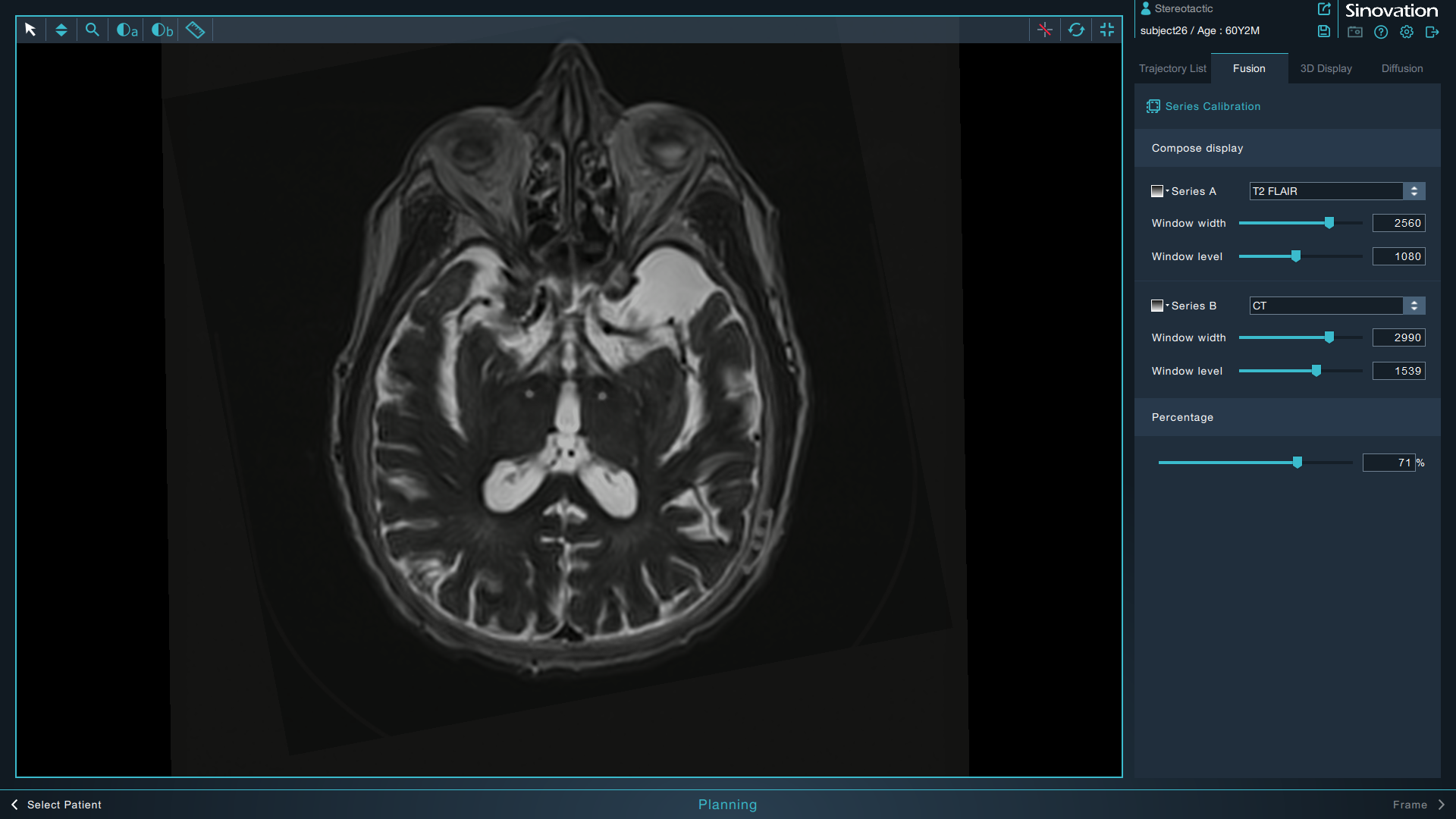

Supplement: Supplementary file 1 [file Data_Sheet_1.ZIP › Postoperative electrode position/subject26.png]

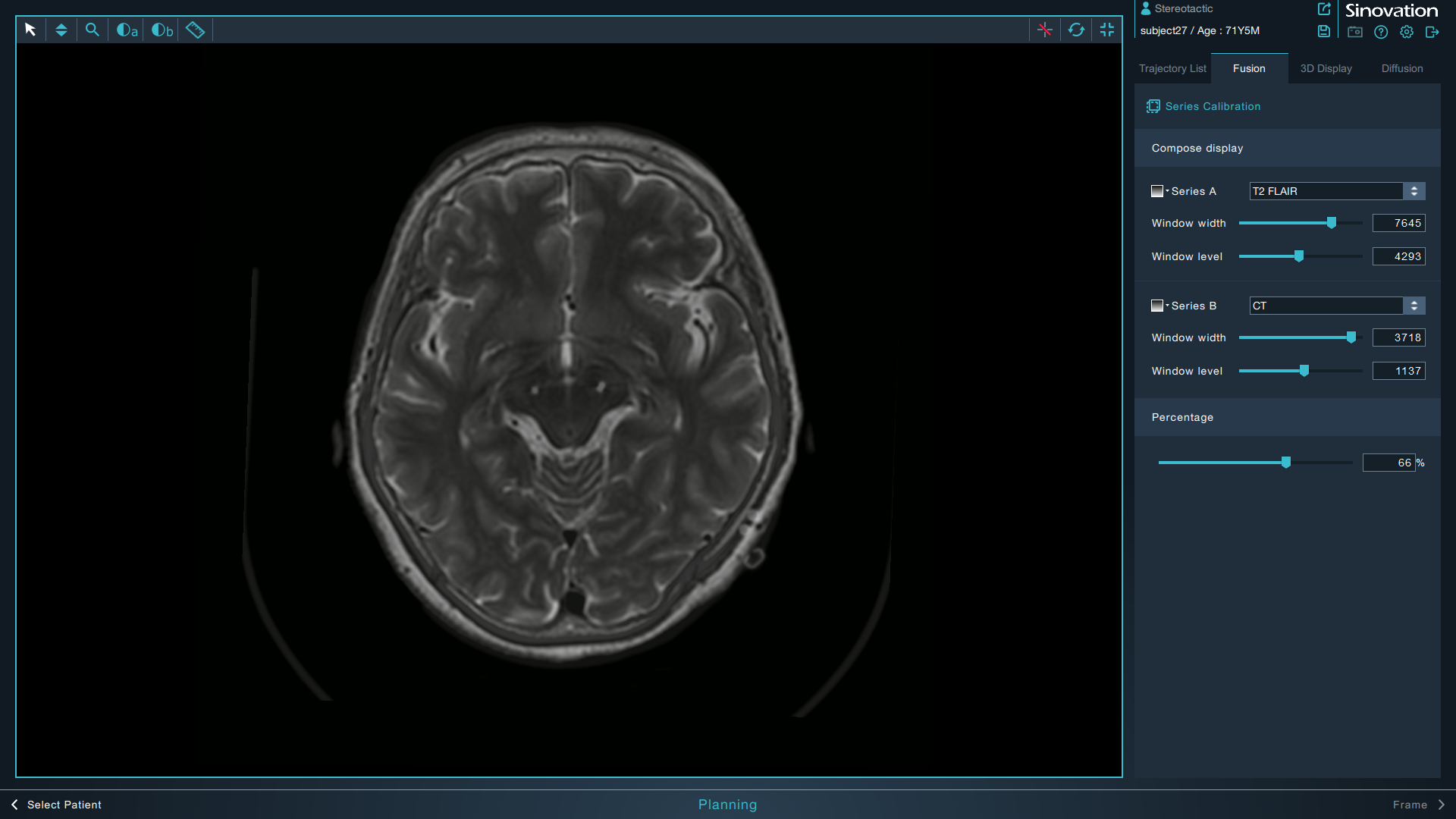

Supplement: Supplementary file 1 [file Data_Sheet_1.ZIP › Postoperative electrode position/subject27.png]

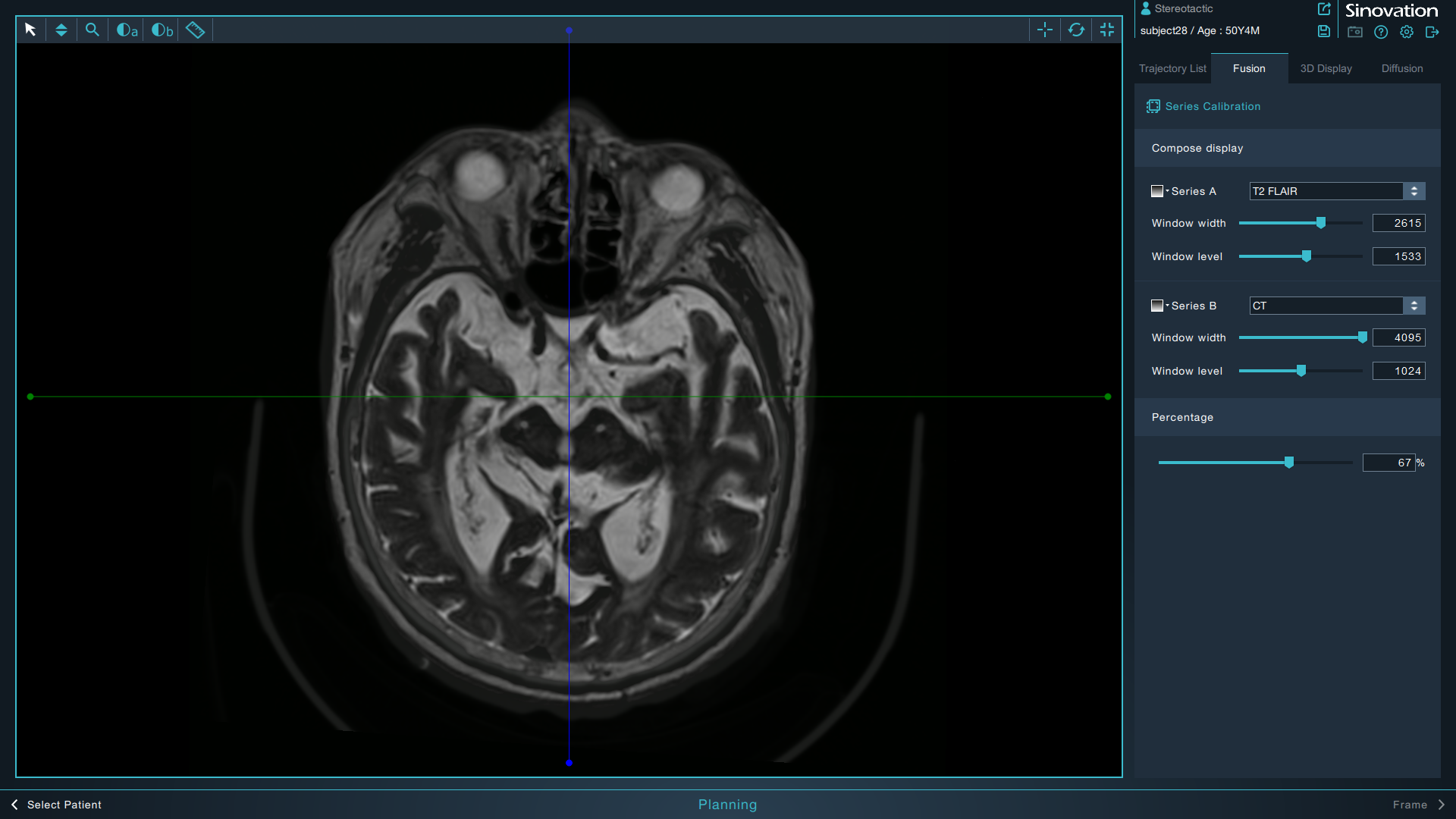

Supplement: Supplementary file 1 [file Data_Sheet_1.ZIP › Postoperative electrode position/subject28.png]

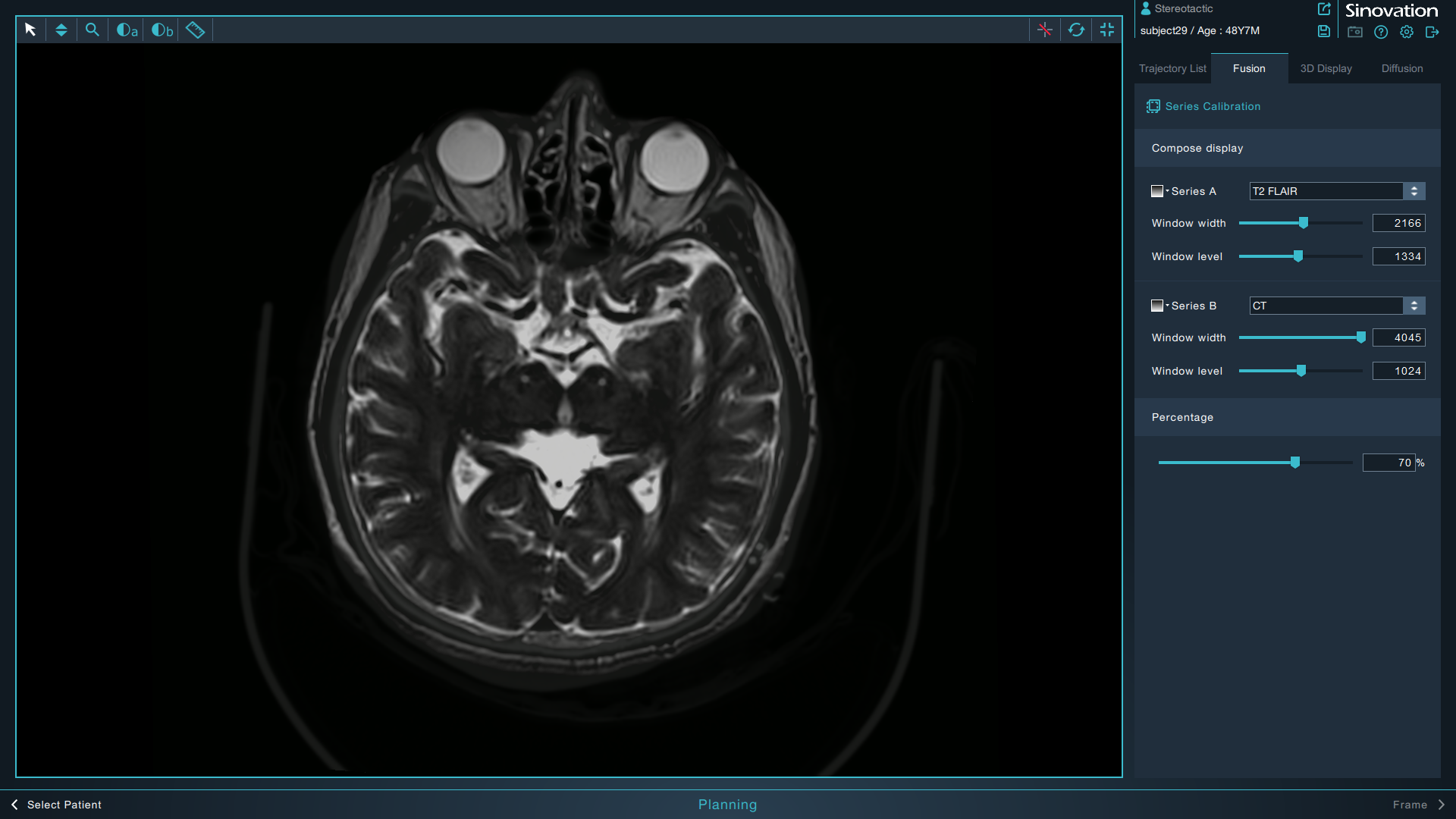

Supplement: Supplementary file 1 [file Data_Sheet_1.ZIP › Postoperative electrode position/subject29.png]

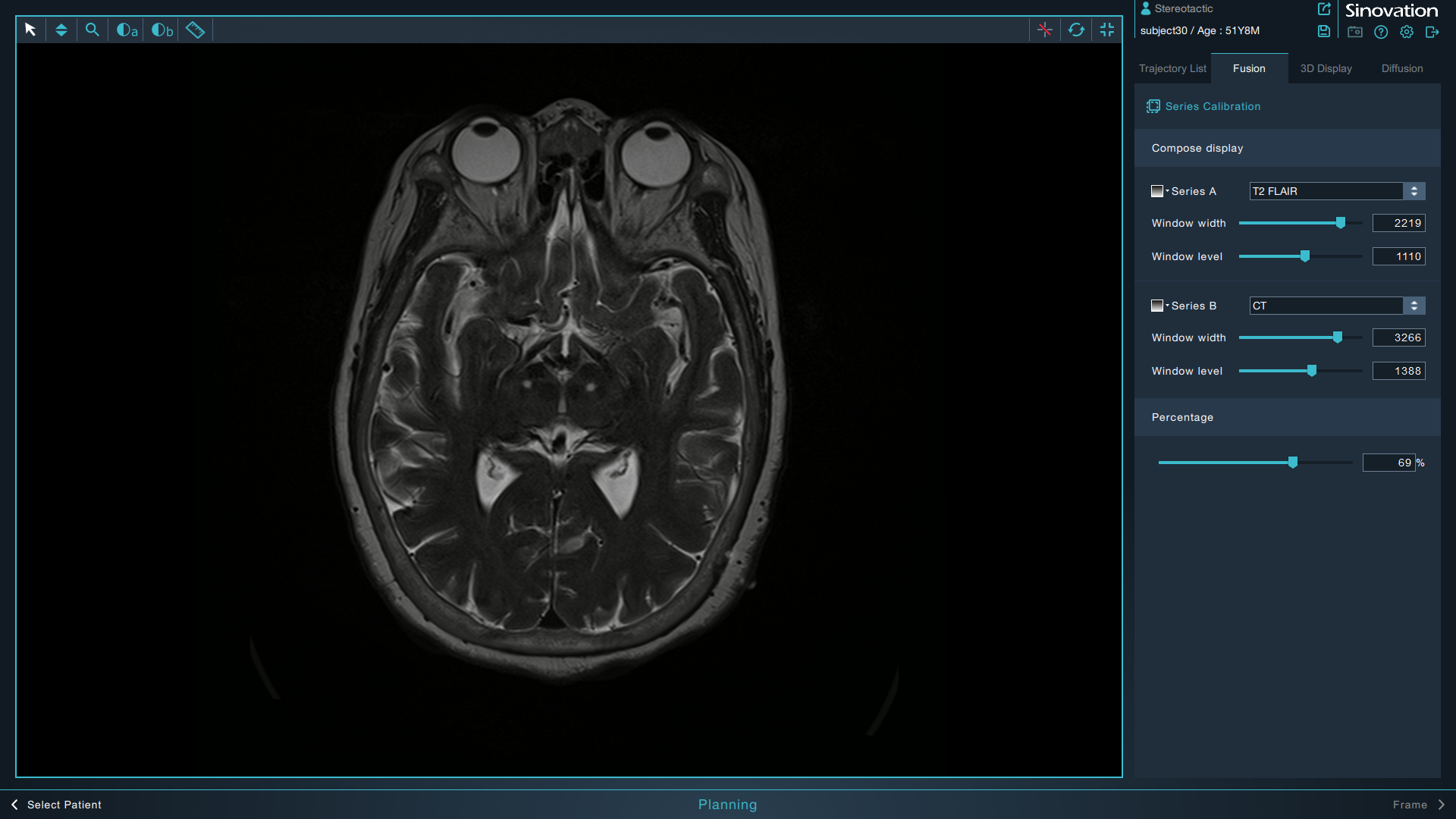

Supplement: Supplementary file 1 [file Data_Sheet_1.ZIP › Postoperative electrode position/subject30.png]
